# Cobalt(II) Aqua Complex-Mediated Hydrogen Peroxide Activation: Possible Roles of HOOOH and Co(II)–OOOH Intermediates in Singlet Oxygen Generation

**DOI:** 10.1021/acs.inorgchem.4c03966

**Published:** 2024-12-25

**Authors:** Hsing-Yin Chen, Yu-Fen Lin

**Affiliations:** Department of Medicinal and Applied Chemistry, Kaohsiung Medical University, Kaohsiung 80708, Taiwan

## Abstract

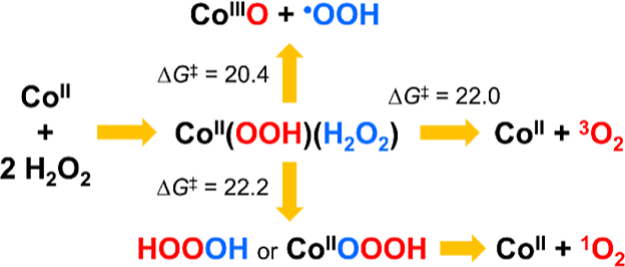

Density functional theory (DFT) calculations indicate that [Co^II^(H_2_O)_6_]^2+^ reacts with two H_2_O_2_ molecules to form [(H_2_O)_4_Co^II^(OOH)(H_2_O_2_)]^+^ reactant complexes, which decompose through three distinct pathways depending on the relative orientation between the coordinated ^–^OOH and H_2_O_2_ ligands. The reactive intermediates produced via these activation pathways include hydroperoxyl (^•^OOH)/superoxide (O_2_^•–^) radicals, singlet oxygen (^1^O_2_), and Co(III) species [(H_2_O)_5_Co^III^(O)]^+^, [(H_2_O)_4_Co^III^(OH)_2_]^+^, and [(H_2_O)_5_Co^III^(OH)]^2+^. The Co(III) species display from moderate to strong oxidizing abilities that have long been overlooked. Remarkably, our DFT calculations reveal the possible formation of hydrogen trioxide (HOOOH) and Co(II)–OOOH intermediates during [(H_2_O)_4_Co^II^(OOH)(H_2_O_2_)]^+^ decomposition and that the hydrolysis of these transient species is a route to ^1^O_2_ production. Because two of the three activation pathways do not involve changes in the oxidation state of the Co center, they may apply to other systems comprising redox-inert metal ions.

## Abstract 2

DFT calculations suggest three pathways of H_2_O_2_ activation by Co(II)_aq_ that generate reactive species including superoxide/hydroperoxyl radical, singlet oxygen, and Co(III) species. One of the most striking findings is the formation of transient species HOOOH and Co(II)–OOOH. The rapid hydrolysis of HOOOH and Co(II)–OOOH is a route to singlet oxygen production.

## 1 Introduction

Hydrogen peroxide (H_2_O_2_) is an eco-friendly oxidant that has long been widely used in daily life and industry. However, H_2_O_2_ itself is not a strong oxidizing agent; it must be activated and converted to more powerful oxygen-containing radicals, such as hydroxyl (^•^OH) and superoxide (O_2_^•–^) radicals, to oxidize substrates. Many transition-metal ions (TMIs) can catalyze H_2_O_2_ decomposition, and TMI-catalyzed H_2_O_2_ activation has been developed as an advanced oxidation process (AOP) for water treatment and environmental remediation.^1−14^ Recently, AOPs have been applied to chemodynamic therapy for cancer treatment.^15−20^ Additionally, because H_2_O_2_ is a cellular metabolism byproduct and many TMIs are essential micronutrients for life, TMIs inevitably react with H_2_O_2_ in living organisms and play roles in biological oxidation processes.^21−23^

Usually, TMI-catalyzed H_2_O_2_ activation involves redox between the TMI and H_2_O_2_. A representative example is the Fenton reaction, which uses the iron(II) aqua complex [Fe^II^(H_2_O)_6_]^2+^ catalyst. In a typical Fenton reaction, H_2_O_2_ first oxidizes iron(II) to iron(III), generating ^•^OH and OH^–^ (reaction 1) and then reduces iron(III) back to iron(II), generating hydroperoxyl radicals (^•^OOH) and H^+^ (reaction 2). However, the Fenton reaction is complex and generates high-valent iron(IV)–oxo species (reaction 3).
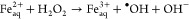
1
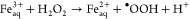
2
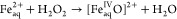
3

In Fenton (with only water ligands) and Fenton-like (with ligands other than water) reactions, the predominant formation of ^•^OH or iron(IV) species is determined by various factors, including pH conditions,^24−28^ buffer systems,^26,29^ and ligands.^28,30^

Previous studies have indicated that Co(II)/H_2_O_2_ mixtures can damage DNA.^31−33^ In recent years, although Co(II) ions have been extensively used in organic pollutant AOPs,^13,14,34−39^ our understanding of the Co(II)/H_2_O_2_ reaction mechanism is far behind that of the Fe(II)/H_2_O_2_ reaction mechanism. The cobalt(II) aqua complex [Co^II^(H_2_O)_6_]^2+^ can demonstrably catalyze H_2_O_2_ decomposition, although not as efficiently as [Fe(H_2_O)_6_]^2+^.^13,14,31−34,36,38,40−42^ However, the standard electrode potential of Co^3+^/Co^2+^ is 1.920 V, which is much higher than that of Fe^3+^/Fe^2+^ (0.771 V). Because the standard electrode potential of H_2_O_2_,H^+^/^•^OH,H_2_O is 0.710 V, the Co^2+^_aq_-mediated Fenton-like reaction (analogous to reaction 1) should be highly endergonic by 28 kcal/mol and, therefore, very unlikely to occur. In fact, previous studies have reported that the cobalt ion’s oxidation state did not change during the reaction between Co^2+^_aq_ and H_2_O_2_.^31,41^ In addition, in contrast to the Fe^2+^_aq_/H_2_O_2_ reaction, where ^•^OH and Fe(IV) species are the major reactive oxidants, the reaction between Co^2+^_aq_ and H_2_O_2_ reportedly generates distinct reactive species, including superoxide (O_2_^•–^),^14,34,40−42^ singlet oxygen (^1^O_2_),^32,33^^•^OH,^14,32−34,40,42^ and crypto-^•^OH (a reactive species possessing a reactivity similar to that of ^•^OH but much less sensitive to inhibition by certain ^•^OH scavengers).^31,32,43^ Furthermore, Co(II)-mediated AOPs display the maximal efficiency under neutral to basic conditions,^13,34,35,38^ as opposed to the optimal conditions (pH 2–3) for Fe(II)-mediated AOPs,^4,9,10^ suggesting different mechanisms for Co^2+^_aq_- and Fe^2+^_aq_-catalyzed H_2_O_2_ decompositions.

Shul’pin et al.^44−47^ used H_2_O_2_ to oxidize hydrocarbons, catalyzed using aqua complexes of redox-inert metal ions, including Al^3+^, Be^2+^, Zn^2+^, and Cd^2+^,^44,45^ and proposed that these catalyzed H_2_O_2_ decompositions involve two H_2_O_2_ molecules and occur through the following mechanism (reactions 4–7):^46,47^
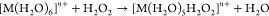
4
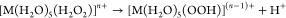
5
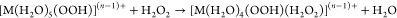
6
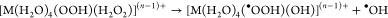
7

The first step is the substitution of H_2_O_2_ for a H_2_O ligand (reaction 4), followed by the deprotonation of the coordinated H_2_O_2_ to form the hydroperoxo complex [M(H_2_O)_5_(OOH)]^(*n*−1)+^ (reaction 5). Then, a second H_2_O_2_ molecule replaces the other H_2_O ligand, generating the [M(H_2_O)_4_(OOH)(H_2_O_2_)]^(*n*−1)+^ complex (reaction 6). Next, in the coordinated H_2_O_2_, the O–O bond is reductively cleaved, which is triggered by electron transfer from the ^–^OOH ligand, forming ^•^OH and hydroperoxyl (^•^OOH) radicals (reaction 7). Notably, in this mechanism, because H_2_O_2_ is reduced by a coligand (^−^OOH) rather than metal ions, the oxidation state of the metal ions does not change. However, this reaction mechanism appears to lack a thermodynamic driving force, as the density functional theory (DFT) calculations showed that the overall reaction was from moderately to highly endergonic.^46,47^ In addition, because the transition states of the crucial activation step (reaction 7) are missing in these previous DFT studies, the overall activation energies have not been determined.^46,47^

Meyerstein et al.^48^ recently used spectroscopy to investigate the Co^2+^_aq_/H_2_O_2_ reaction kinetics and found that the reaction involved three equilibrium processes, which were assigned to three consecutive H_2_O_2_-for-water-ligand substitutions. Building upon work conducted by Shul’pin et al.,^46^ Meyerstein et al.^47^ proposed that Co^2+^_aq_-mediated H_2_O_2_ decomposition proceeded via the formation of the Co^II^(H_2_O)_3_(OOH)_2_(H_2_O_2_) complex, followed by reductive HO–OH bond cleavage to produce Co^II^(H_2_O)_3_(OOH)(^•^OOH)(OH) and ^•^OH. The DFT calculations indicated that although Co^II^(H_2_O)_4_(OOH)(H_2_O_2_) decomposition was endergonic, Co^II^(H_2_O)_3_(OOH)_2_(H_2_O_2_) decomposition became exergonic because the unpaired electron delocalized over both OOH ligands. Nevertheless, previous DFT calculations did not provide any information about transition states and, thus, reaction kinetics.^48^ Notably, neither Shul’pin et al.’s nor Meyerstein et al.’s proposed mechanism accounts for singlet oxygen formation during the reaction between Co^2+^_aq_ and H_2_O_2_.

Therefore, we herein present a DFT analysis of [Co^II^(H_2_O)_6_]^2+^-catalyzed H_2_O_2_ decomposition involving two H_2_O_2_ molecules. Three distinct reaction pathways leading to the formations of ^•^OOH/O_2_^•–^, Co(III) species, and ^1^O_2_ were discovered. All these reaction pathways are thermodynamically favorable, with accessible activation energies of 20–22 kcal/mol. Remarkably, our DFT calculations reveal that ^1^O_2_ production is derived from the hydrolysis of hydrogen trioxide (HOOOH) and/or the cobalt(II)–hydrotrioxide complex (Co(II)–OOOH). The hydrogen atom abstraction reactivities of Co(III) species are also evaluated.

## 2 Benchmark Study

To choose a reliable computational approach for this study, we first conducted a benchmark study of DFT functionals and implicit solvation models for the standard electrode potential (*E*°) of Co^3+^/Co^2+^. The theoretical *E*°(Co^3+^/Co^2+^) value was obtained by subtracting the absolute potential of the standard hydrogen electrode (4.44 V) from the calculated free-energy change for the reduction from [Co(H_2_O)_6_]^3+^ to [Co(H_2_O)_6_]^2+^ as follows:
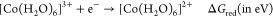
8
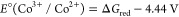
9

The calculated *E*°(Co^3+^/Co^2+^) values are summarized in Table 1. These results show that the solvation model influences the calculated electrode potentials more strongly than the DFT functional. Specifically, calculations performed using the conductor-like polarizable continuum model (CPCM) solvation model substantially overestimate *E*°(Co^3+^/Co^2+^); regardless of which DFT functionals are used, the errors are in the range 1.60–2.08 V. By contrast, calculations performed using the density-based solvation model (SMD) provide more satisfactory results, with errors of 0.26–0.79 V. Among the evaluated DFT functionals, TPSSh showed the best performance.

**Table 1:** Standard Electrode Potentials Calculated for Co(III)/Co(II)^a^

	CPCM	SMD
B3LYP	3.84	2.51
PBE0	3.75	2.43
PW6B95	3.95	2.71
MN15	3.90	2.63
BMK	4.00	2.64
TPSSh	3.52	2.18
expt		1.92

a Def2-TZVP basis sets are used in the calculations.

## 3 Computational Methods

According to the benchmark study results, the TPSSh functional^49,50^ and triple-ζ valence quality Def2-TZVP basis sets were used in the main study. The geometries were optimized and vibrational frequencies were calculated for the aqueous environment treated using the SMD solvation model.^51^ All the optimized structures were verified to possess no and one imaginary vibrational frequency for local minima and transition states, respectively. To confirm the proper connection to the corresponding intermediates, intrinsic reaction coordinates (IRCs) were calculated for each transition-state structure. To correct the solution entropy error introduced using gas-phase statistical thermodynamic formulas in standard quantum chemical calculations, the absolute entropies of 1 M solutes in water and 55.5 M water were estimated using eqs 10 and 11, respectively, as follows:^30^
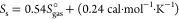
10
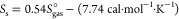
11

The theoretical p*K*_a_ values of the cobalt complexes were evaluated based on the linear regression presented in eq 12 as follows:
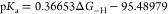
12where Δ*G*_–H_ is the free-energy difference between the conjugate base and acid, as computed using the SMD/TPSSh/Def2-TZVP method. The training set of this linear regression includes Fe(III), Cr(III), Sc(III), Cu(II), Fe(II), Co(II), and Mn(II) aqua complexes selected because they possess less-scattered experimental p*K*_a_ values (Table S1). Equation 12 can be used to accurately reproduce the p*K*_a_ values of first-row transition-metal aqua complexes, including the oxovanadium(IV) complex ([V^IV^(H_2_O)_5_(O)]^2+^) (Table S2).

The singlet oxygen (^1^O_2_) energy (^1^Δ_g_) was calculated using the approximate spin-projection (AP) method.^52^ The spin-projected energy of the singlet oxygen was evaluated using eqs 13 and 14a,14b as follows:
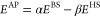
13
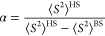
14a
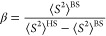
14bwhere *E*^BS^ is the energy of the broken-symmetry open shell’s singlet state, and *E*^HS^ is the energy of the high-spin triplet state.

Because the investigated mechanisms involve protonation and deprotonation, the corresponding energies are pH dependent and determined using eq 15 as follows:
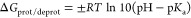
15All the calculations were accomplished using the Gaussian 16 program.^53^

## 4 Results and Discussion

### 4.1 Ligand Exchanges between [Co^II^(H_2_O)_6_]^2+^ and H_2_O_2_

**Figure 1:**
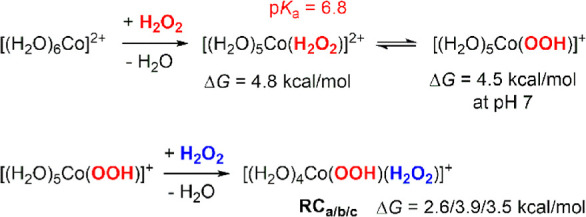
Ligand exchanges between [Co^II^(H_2_O)_6_]^2+^ and H_2_O_2_.

[Co^II^(H_2_O)_6_]^2+^-mediated H_2_O_2_ activation begins with the substitution of H_2_O_2_ for H_2_O ligands. The first substitution, forming [(H_2_O)_5_Co^II^(H_2_O_2_)]^2+^, was calculated as endergonic by 4.8 kcal/mol (Figure 1). As H_2_O_2_ is a weaker base than H_2_O, this result is expected. For [(H_2_O)_5_Co^II^(H_2_O_2_)]^2+^, the coordinated H_2_O_2_ was predicted to possess a p*K*_a_ of 6.8, suggesting that both the protonated and deprotonated forms {[(H_2_O)_5_Co^II^(H_2_O_2_)]^2+^ and [(H_2_O)_5_Co^II^(OOH)]^+^, respectively} coexist at a neutral pH. Then, [(H_2_O)_5_Co^II^(OOH)]^+^ undergoes a second ligand exchange reaction with H_2_O_2_ to generate the reactant complex (**RC**) *cis*-[(H_2_O)_4_Co^II^(OOH)(H_2_O_2_)]^+^, where ^–^OOH and H_2_O_2_ are oriented in a cis-conformation. Because of the ^–^OOH anionic ligand, the second ligand exchange reaction was somewhat less endergonic than the first (Figure 1). According to the relative orientation between ^–^OOH and H_2_O_2_, the *cis*-[(H_2_O)_4_Co^II^(OOH)(H_2_O_2_)]^+^ RC can exhibit three different conformations (**RC**_**a**_, **RC**_**b**_, and **RC**_**c**_), resulting in distinct decomposition pathways that are described in the following section. Because the *trans*-[(H_2_O)_4_Co^II^(OOH)(H_2_O_2_)]^+^ was identified at 1.8 kcal/mol higher than the cis-conformer and it's decomposition possesses a higher energy barrier, the results of *trans*-[(H_2_O)_4_Co^II^(OOH)(H_2_O_2_)]^+^ were discussed in Supporting Information (Figure S2).

### 4.2 Decomposition Pathways of *cis*-[(H_2_O)_4_Co^II^(OOH)(H_2_O_2_)]^+^

**Figure 2:**
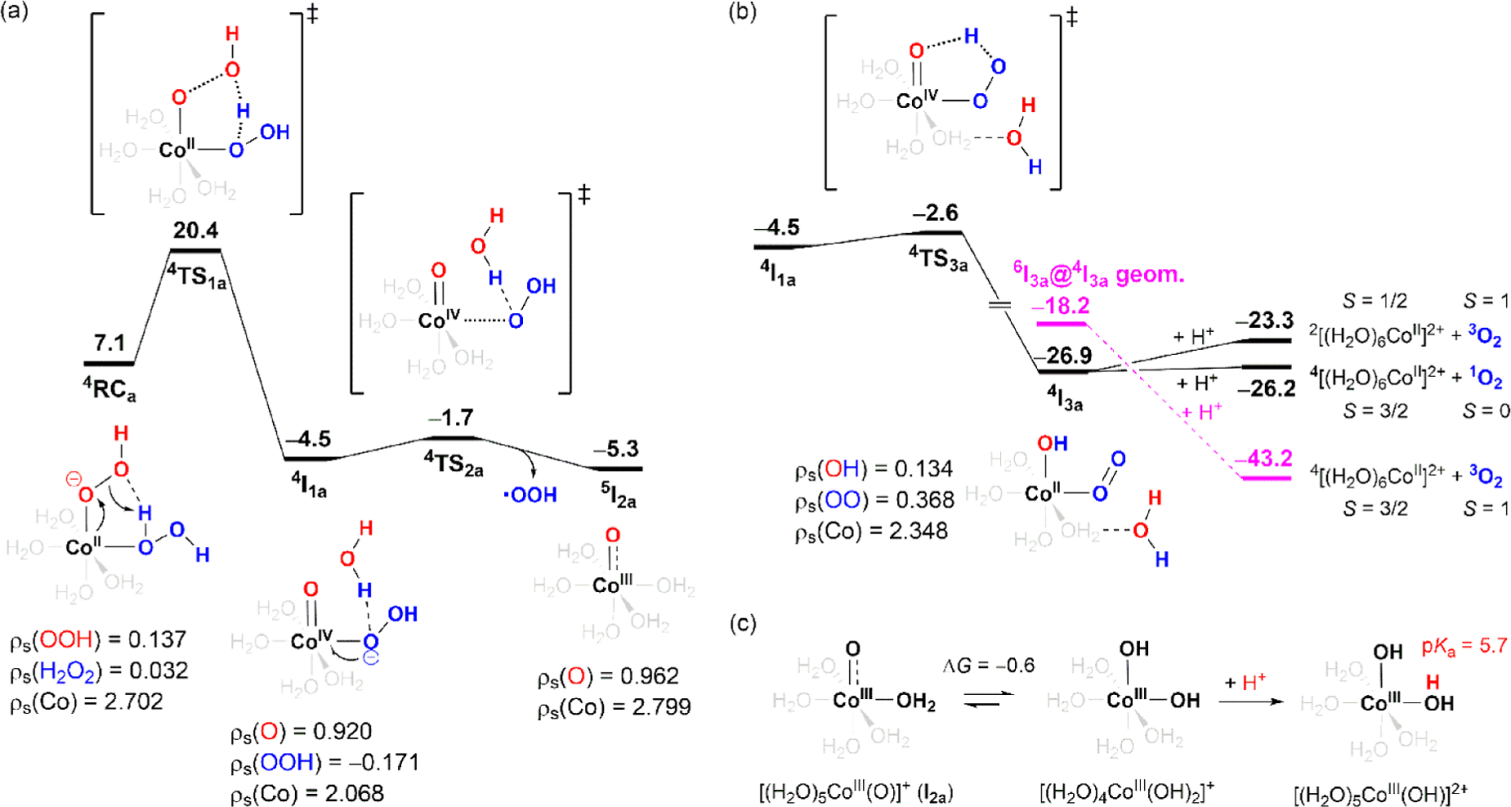
(a,b) Free-energy profile of [(H_2_O)_4_Co^II^(OOH)(H_2_O_2_)]^+^ decomposition assisted by proton transfer from H_2_O_2_ to ^–^OOH. (c) Co(III) speciation. The energy unit is kcal/mol.

**Figure 3:**
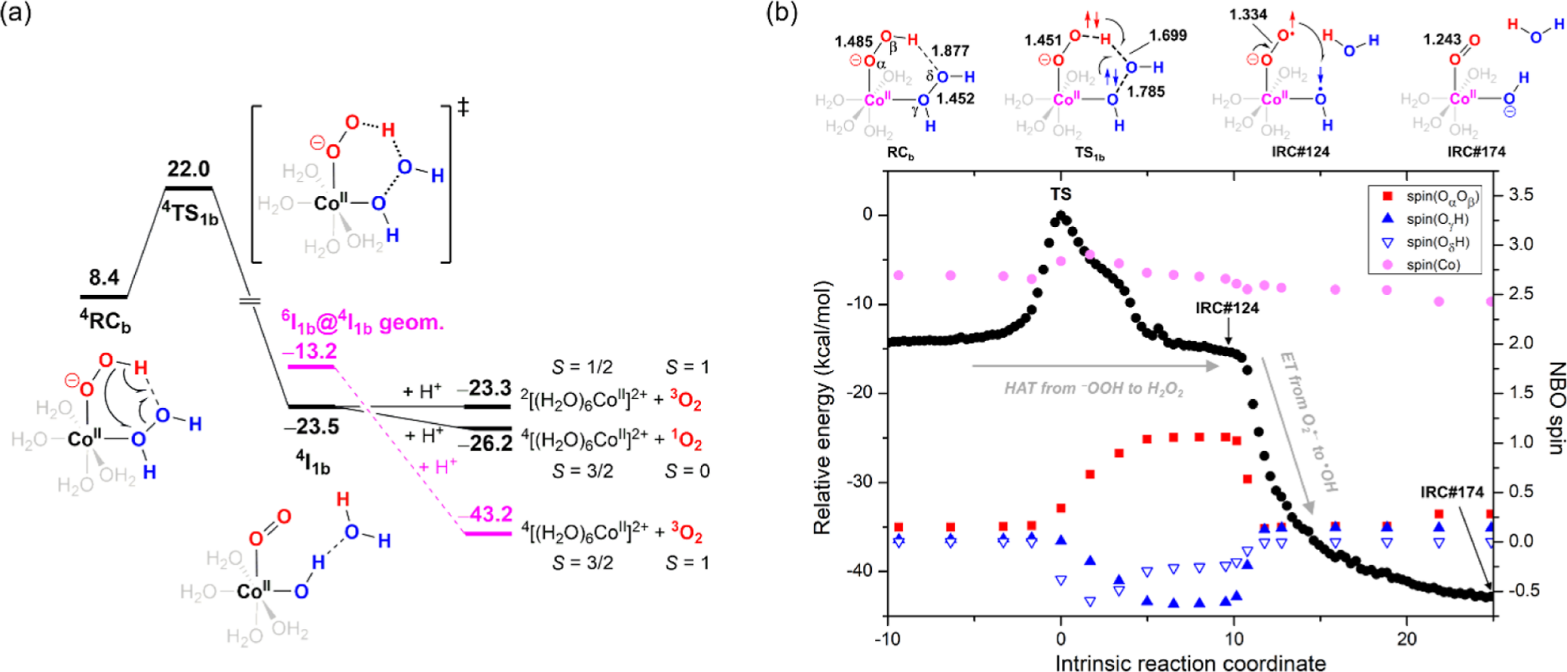
(a) Free-energy profile for [(H_2_O)_4_Co^II^(OOH)(H_2_O_2_)]^+^ decomposition via hydrogen atom-coupled electron transfer from ^–^OOH to H_2_O_2_. (b) Spin-density evolution along the IRC path of **TS**_**1b**_. The energy unit is kcal/mol.

**Figure 4:**
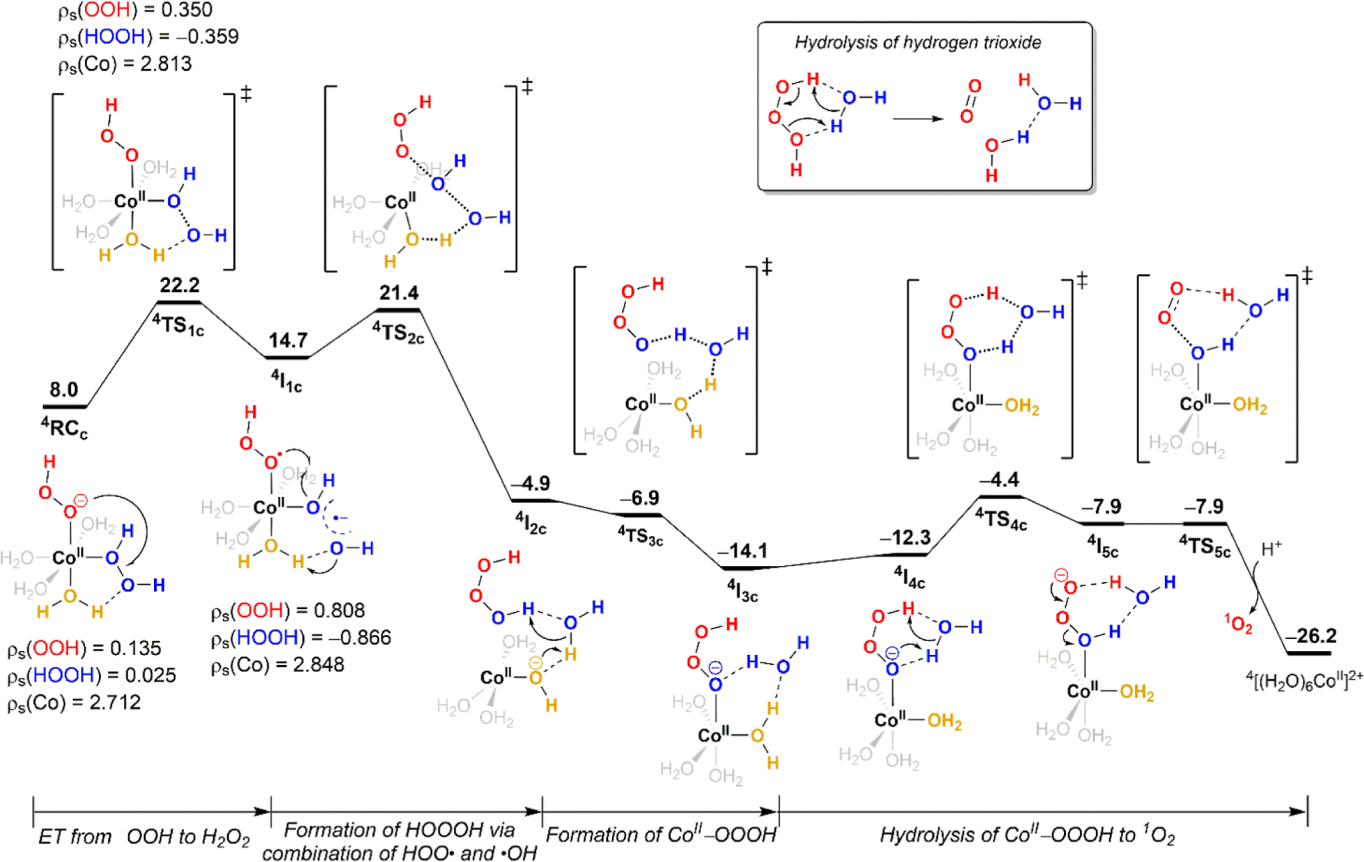
Free-energy profile for [(H_2_O)_4_Co^II^(OOH)(H_2_O_2_)]^+^ decomposition via electron transfer from ^–^OOH to H_2_O_2_. The energy unit is kcal/mol.

Before discussing the H_2_O_2_ activation mechanisms, consider that the formation energy of the [(H_2_O)_4_Co^II^(OOH)(H_2_O_2_)]^+^ RC is pH dependent because it involves deprotonation, as shown in Figure 1. Consequently, the free-energy changes along the subsequent decomposition pathways are also pH dependent. The relative free energies of the following free-energy profiles are evaluated under neutral conditions (pH 7) (Figures 2–4). According to eq 15, these values will decrease/increase by 1.36 kcal/mol per unit of increasing/decreasing pH. Furthermore, for each reaction pathway, the high- and low-spin states (quartet and doublet, respectively) were calculated. The high spin-state pathways were at apparently lower energies than the low spin-state pathways (Figures S3–S5); only the free-energy profiles of the high-spin states are presented herein.

#### 4.2.1 Decomposition via Proton Transfer from H_2_O_2_ to ^–^OOH

This reaction pathway starts from **RC**_**a**_, where H_2_O_2_ donates a proton to form a hydrogen bond with the distal oxygen atom in ^–^OOH (Figure 2a). Then, in ^–^OOH, the O–OH bond is heterolytically cleaved, as assisted by the proton transferred from the H_2_O_2_ to the leaving group (^−^OH), forming the Co(IV)–oxo–hydroperoxo complex ^4^[(H_2_O)_4_Co^IV^(O)(OOH)]^+^·H_2_O (^**4**^**I**_**1a**_). For ^**4**^**I**_**1a**_, population analysis indicates that the OOH moiety possesses a low spin density (−0.171) and, therefore, should be considered as ^–^OOH rather than ^•^OOH. In addition, the Co–O bond is characteristically short (1.62 Å), which is comparable to Fe–O bonds in well-known Fe(IV)–oxo complexes.^27,29,30,54−56^ According to these results, in ^**4**^**I**_**1a**_, we assigned the formal oxidation state of the Co to +4. However, this Co(IV) complex is unstable and immediately reduced back to Co(III) or Co(II) via two distinct pathways. The first possibility is that the Co(IV) in ^**4**^**I**_**1a**_ is reduced by an electron transfer from the ^–^OOH ligand, generating the Co(III)–oxo/oxyl complex ^5^[(H_2_O)_5_Co^III^(O)]^+^ (^**5**^**I**_**2a**_) accompanied by the release of the ^•^OOH radical (Figure 2a). ^**5**^**I**_**2a**_ possesses a relatively longer Co–O bond (1.70 Å) than ^**4**^**I**_**1a**_, suggesting that compared with the terminal oxygen atom in ^**4**^**I**_**1a**_, that in ^**5**^**I**_**2a**_ is more oxyl-like. For comparison, the iron(III) analogue ^6^[(H_2_O)_5_Fe^III^(O)]^+^ was calculated and the Fe–O bond length was also 1.70 Å.

Alternatively, the Co(IV) in ^**4**^**I**_**1a**_ can be reduced back to Co(II) via a hydrogen atom transfer from ^–^OOH to oxo group coupled an electron transfer from the resulting O_2_^•–^ to Co(III), generating the dioxygen complex ^4^[(H_2_O)_4_Co^II^(O_2_)(OH)]^+^·H_2_O (^**4**^**I**_**3a**_, see Figure 2b). The latter process [i.e., electron transfer from O_2_^•–^ to Co(III)] can be evidenced by the fact that the coordinated dioxygen in ^**4**^**I**_**3a**_ possesses 0.368 spin density, which is considerably smaller than that should be possessed by O_2_^•–^. According to Wigner spin conservation rule, ^**4**^**I**_**3a**_ can decay to ^2^[(H_2_O)_6_Co^II^]^2+^ + ^3^O_2_ or ^4^[(H_2_O)_6_Co^II^]^2+^ + ^1^O_2_; however, both processes are slightly endergonic as shown in Figure 2b. On the other hand, the dioxygen complex **I**_**3a**_ can also exist in a sextet state in which high spin Co(II) ferromagnetically coupled to triplet oxygen. Single-point-energy calculation for the sextet state ^**6**^**I**_**3a**_ at geometry of ^**4**^**I**_**3a**_ (denoted by ^**6**^**I**_**3a**_@^**4**^**I**_**3a**_ in Figure 2b) reveals that it lies 8.7 kcal/mol above ^**4**^**I**_**3a**_. In addition, we have tried to optimize [(H_2_O)_4_Co^II^(O_2_)(OH)]^+^·H_2_O complex (**I**_**3a**_) at sextet state. However, geometry optimization of ^**6**^**I**_**3a**_ spontaneously converges to a fragmented structure in which ^3^O_2_ already dissociated from Co(II), implying a repulsive interaction between high spin Co(II) and ^3^O_2_ in ^**6**^**I**_**3a**_. Based on these results, it is very likely that along the O_2_ dissociation coordinate of ^**4**^**I**_**3a**_ the sextet-state energy continues to decline and crossover the quartet state and, therefore, ^**4**^**I**_**3a**_ can decay to ^4^[(H_2_O)_6_Co^II^]^2+^ + ^3^O_2_ as main product, which is most thermodynamically favorable, through spin transition at crossing point caused by spin–orbit coupling.

[(H_2_O)_4_Co^III^(OH)_2_]^+^ is another tautomer of the [(H_2_O)_5_Co^III^(O)]^+^ complex (the species produced by reaction in Figure 2a). Surprisingly, [(H_2_O)_4_Co^III^(OH)_2_]^+^ was calculated at only 0.6 kcal/mol lower than [(H_2_O)_5_Co^III^(O)]^+^ (Figure 2c). For comparison, the iron(III) counterparts, [(H_2_O)_5_Fe^III^(O)]^+^ and [(H_2_O)_4_Fe^III^(OH)_2_]^+^, respectively, were calculated, and the latter was 14.3 kcal/mol lower than the former, revealing that in aqueous solutions, Co(III) and Fe(III) speciations are very different. Very recently, Cao et al. used confocal Raman spectroscopy to investigate an aqueous Co(III) solution and detected a characteristic vibrational band at 835 cm^–1^, which was assigned to the Co(III)–O vibration of the Co(III)–oxo species.^57^ Our DFT calculations confirm this spectral assignment: In [(H_2_O)_5_Co^III^(O)]^+^, the Co(III)–O vibrational frequency was calculated at 792 and 831 cm^–1^, using the SMD/TPSSh/Def2-TZVP and SMD/PBE0/Def2-TZVP methods, respectively. The Raman spectroscopy study by Cao et al. and our DFT calculations establish the existence of Co(III)–oxo/oxyl species in aqueous Co(III) solutions. Moreover, under slightly acidic conditions, [(H_2_O)_4_Co^III^(OH)_2_]^+^ was predicted to be protonated to form the conjugate acid [(H_2_O)_5_Co^III^(OH)]^2+^ (p*K*_a_ = 5.7, Figure 2b). As will be discussed in the following sections, the Co(III) [(H_2_O)_5_Co^III^(O)]^+^, [(H_2_O)_4_Co^III^(OH)_2_]^+^, and [(H_2_O)_5_Co^III^(OH)]^2+^ species exhibit different oxidizing abilities.

#### 4.2.2 Decomposition via Hydrogen Atom-Coupled Electron Transfer from ^–^OOH to H_2_O_2_

This reaction pathway starts from ^**4**^**RC**_**b**_, where ^–^OOH donates a proton to form a hydrogen bond with the distal oxygen atom in H_2_O_2_ (Figure 3a), and involves the two-electron reduction of H_2_O_2_. Initially, in H_2_O_2_, the HO–OH bond is homolytically cleaved; meanwhile, the leaving ^•^OH radical abstracts the hydrogen atom from ^–^OOH (the first reduction), forming the transient species [(H_2_O)_4_Co^II^(O_2_^•–^)(^•^OH)]^+^·H_2_O; thereupon, O_2_^•–^ transfers an electron to ^•^OH (the second reduction), forming the dioxygen complex [(H_2_O)_4_Co^II^(O_2_)(OH)]^+^·H_2_O (^**4**^**I**_**1b**_). This two-electron reduction can be evidenced by monitoring the evolution of the spin-density population along the IRC path of transition state ^**4**^**TS**_**1b**_, as shown in Figure 3b. Clearly, from ^**4**^**RC**_**b**_ to ^**4**^**TS**_**1b**_ to IRC-point 124, the α-spin density of the OO moiety in the ^–^OOH ligand increases from 0.148 to 1.061 (solid red squares in Figure 3b), and the β-spin density of the coordinated OH increases from 0.024 to −0.609 (solid blue triangles in Figure 3b), indicating that a hydrogen atom (with β electron) transfers from ^–^OOH to H_2_O_2_. Then, the α- and β-spin densities of the coordinated O_2_^•–^ and ^•^OH, respectively, suddenly both decrease to nearly zero, accompanied by a drastic energy drop, revealing an α-electron transfer from O_2_^•–^ to ^•^OH. Accordingly, although ^**4**^**RC**_**b**_ decomposes to ^**4**^**I**_**1b**_ via a concerted mechanism (i.e., through only one transition state), this should be regarded as a hydrogen atom transfer followed by an electron transfer from ^–^OOH to H_2_O_2_. Additionally, during this decomposition, the spin density of the Co center negligibly changes (solid pink circles in Figure 3b), implying that the change in the Co(II) oxidation state is not involved in this H_2_O_2_ activation pathway. Because ^**4**^**I**_**1b**_ is the same dioxygen complex as ^**4**^**I**_**3a**_ in Figure 2b, only different in configuration, it is thus expected to decay to ^4^[Co^II^(H_2_O)_6_]^2+^ + ^3^O_2_ as main product (Figure 3a) according to the reason mentioned above.

#### 4.2.3 Decomposition via Electron Transfer from ^–^OOH to H_2_O_2_

**Figure 5:**
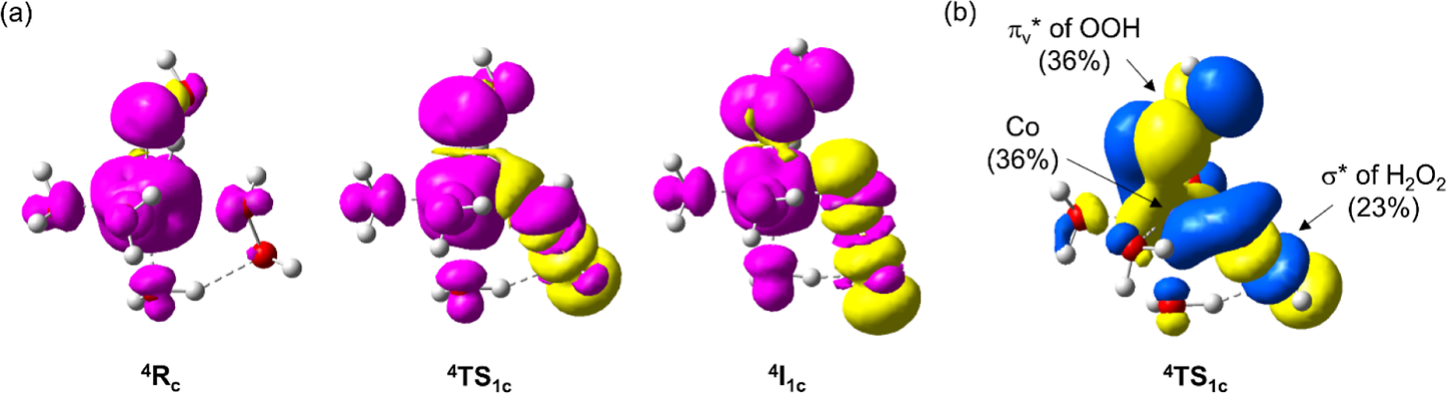
(a) Spin–density plots of **R**_**c**_, **TS**_**1c**_, and **I**_**1c**_. (b) β-HOMO of **TS**_**1c**_.

This reaction pathway proceeds through an electron transfer from ^–^OOH to H_2_O_2_, reductively cleaving the HO–OH bond (from ^**4**^**RC**_**c**_ to ^**4**^**I**_**1c**_ in Figure 4). The evidence supporting the hypothesis that H_2_O_2_ is reduced by ^–^OOH rather than Co(II) is the substantial spin-density change in OOH from 0.135 to 0.350 and 0.808 at ^**4**^**RC**_**c**_, ^**4**^**TS**_**1c**_, and ^**4**^**I**_**1c**_, respectively, whereas the Co spin density only slightly changes from 2.712 to 2.848 during this process. More specifically, the electron is transferred from π_v_* in ^–^OOH to σ* in H_2_O_2_, as can be clearly seen from spin–density plots of ^**4**^**RC**_**c**_, ^**4**^**TS**_**1c**_, and ^**4**^**I**_**1c**_, and the highest occupied molecular orbital (β-HOMO) of ^**4**^**TS**_**1c**_ depicted in Figure 5. Population analysis reveals that Co has a significant contribution (36%) to β-HOMO of ^**4**^**TS**_**1c**_, suggesting that this electron transfer is mediated via the assistance of Co. In ^**4**^**I**_**1c**_, the H_2_O_2_ O–O bond is partially broken, at 2.182 Å, and the excess spin and negative charge are delocalized between both OH groups. The dissociation of ^**4**^**I**_**1c**_ into [(H_2_O)_5_Co^II^(^•^OOH)(OH)]^+^ + ^•^OH was calculated as further endergonic by 3.1 kcal/mol. Thus, the overall endergonicity of [Co^II^(H_2_O)_6_]^2+^ + 2H_2_O_2_ → [(H_2_O)_5_Co^II^(^•^OOH)(OH)]^+^ + ^•^OH + 2H_2_O + H^+^ (analogous to reactions 4–7 in Shul’pin et al.’s proposed mechanism for redox-inert metal ion-mediated H_2_O_2_ activation) is 17.8 kcal/mol, in line with the previous computational results for Shul’pin et al.’s mechanism.^46,47^

Alternatively, in ^**4**^**I**_**1c**_, the coordinated cis-oriented ^•^OOH and ^•^OH will dissociate and recombine to generate hydrogen trioxide HOOOH (^**4**^**I**_**2c**_). The formation of a HOO–OH bond provides the thermodynamic driving force rendering the reaction as exergonic. Although HOOOH may diffuse into the solution, as HOOOH is generated near cobalt ions it more likely recoordinates with cobalt ions, forming the hydrotrioxide complex Co(II)–OOOH (^**4**^**I**_**3c**_). Subsequently, Co(II)–OOOH undergoes water molecule–assisted proton migration from distal to coordinated oxygen atoms to form the Co(II)–(H)OOO intermediate (^**4**^**I**_**5c**_), which readily decomposes to generate ^1^O_2_ and ^4^[Co^II^(H_2_O)_6_]^2+^. If one of the HOOOH protons is considered as analogous to a cobalt ion, the Co(II)–OOOH hydrolysis (^**4**^**I**_**4c**_ → Co(II) + ^1^O_2_) is analogous to the well-known hydrolysis from HOOOH to ^1^O_2_ in aqueous solutions (see the inset of Figure 4). Although HOOOH decomposition to ^1^O_2_ as main product has been well established,^58^ the presence of Co(II) prevents us from completely ruling out the possibility of ^3^O_2_ production from Co(II)–OOOH hydrolysis.

In atmospheric chemistry, the formation of organic hydrotrioxides (ROOOH) through the combination of ROO^•^ and ^•^OH was first theoretically predicted^59^ and very recently experimentally established.^60^ However, although ^•^OOH and ^•^OH radicals are common intermediates generated during reactions between H_2_O_2_ and metal ions, to the best of our knowledge, the formation of hydrogen trioxide (HOOOH) has never been proposed in Fenton and Fenton-like chemistries. In addition, more recent studies have observed ^1^O_2_ production in reactions between H_2_O_2_ and metal ions.^32,33,61−65^^1^O_2_ production is hypothesized to originate from O_2_^•–^ decay. To the best of our knowledge, our study is the first to propose the possible presence of hydrogen trioxide or metal–hydrotrioxide complexes as precursory intermediates for ^1^O_2_ production during metal ion-mediated H_2_O_2_ activation. More specifically, our DFT calculations indicate that for such a reaction pathway to operate, a redox-inert metal ion is required because redox-inert metal ion–mediated H_2_O_2_ activation involves the formation of *cis*-[(H_2_O)_4_M(OOH)(H_2_O_2_)]^*n*+^ RCs, and the subsequent interligand electron transfer generates ^•^OOH and ^•^OH radicals at a suitable position and orientation poised for combination to form HOOOH. We searched the literature and found only one paper reporting HOOOH production from a metal complex. In that study, the Pt^IV^(PEt_3_)_2_(Cl)(4-tft)(OH)(OOH) complex (4-tft = 4-trifluoromethylphenyl) undergoes photoelimination upon irradiation with 380 nm wavelength light to yield HOOOH and the Pt^II^(PEt_3_)_2_(Cl)(4-tft) complex,^66^ supporting the hypothesis that in metal complexes, cis-oriented ^•^OOH and ^•^OH can combine to yield HOOOH.

### 4.3 Hydrogen Atom Abstraction Reactivity of Aqueous Co(III) Species

A recent study by Cao et al. revealed the formation of Co(III) species during Co(II)-mediated peroxymonosulfate (PMS) activation and that the resulting Co(III) reactive species contributed to micropollutant degradation in Co(II)/PMS.^57^ Our DFT calculations also show the formation of Co(III) species in Co(II)/H_2_O_2_ (Figure 2a). Further, we note that in aqueous solutions, Co(III) species include [(H_2_O)_5_Co^III^(O)]^+^, [(H_2_O)_4_Co^III^(OH)_2_]^+^, and [(H_2_O)_5_Co^III^(OH)]^2+^ (Figure 2c). To assess the oxidizing abilities of these aqueous Co(III) species, hydrogen atom abstraction (H-abst) reactions with isopropanol were calculated (reactions 16–18). Isopropanol was selected as a probe as it is a well-known ^•^OH radical scavenger and widely used in discriminating ^•^OH radicals from other reactive oxygen species in scavenging experiments.
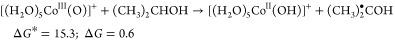
16
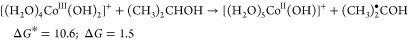
17
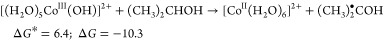
18

The H-abst reactivities of the three Co(III) species were quite different and decreased in the order [(H_2_O)_5_Co^III^(OH)]^2+^ > [(H_2_O)_4_Co^III^(OH)_2_]^+^ > [(H_2_O)_5_Co^III^(O)]^+^. The [(H_2_O)_5_Co^III^(OH)]^2+^ H-abst reaction is characterized by a low activation energy of 6.4 kcal/mol and is exergonic by ∼10 kcal/mol (reaction 18), indicating that [(H_2_O)_5_Co^III^(OH)]^2+^ is a strong oxidant, like ^•^OH radicals, that can activate strong C–H bonds. By contrast, [(H_2_O)_5_Co^III^(O)]^+^ and [(H_2_O)_4_Co^III^(OH)_2_]^+^ display moderate oxidizing abilities, as the corresponding H-abst reactions are slightly endergonic and possess moderate activation energies (reactions 16 and 17, respectively). The computational result showing that [(H_2_O)_5_Co^III^(O)]^+^ possesses the lowest oxidizing ability is consistent with [(H_2_O)_5_Co^III^(O)]^+^ existing long enough in aqueous solutions to be detected using Raman spectroscopy.^57^ Additionally, [(H_2_O)_5_Co^III^(O)]^+^ and [(H_2_O)_4_Co^III^(OH)_2_]^+^ can undergo H-abst like ^•^OH but are much less reactive to the ^•^OH scavenger isopropanol and, therefore, may be the crypto-^•^OH referred to in previous studies.^31,32,43^

**Figure 6:**
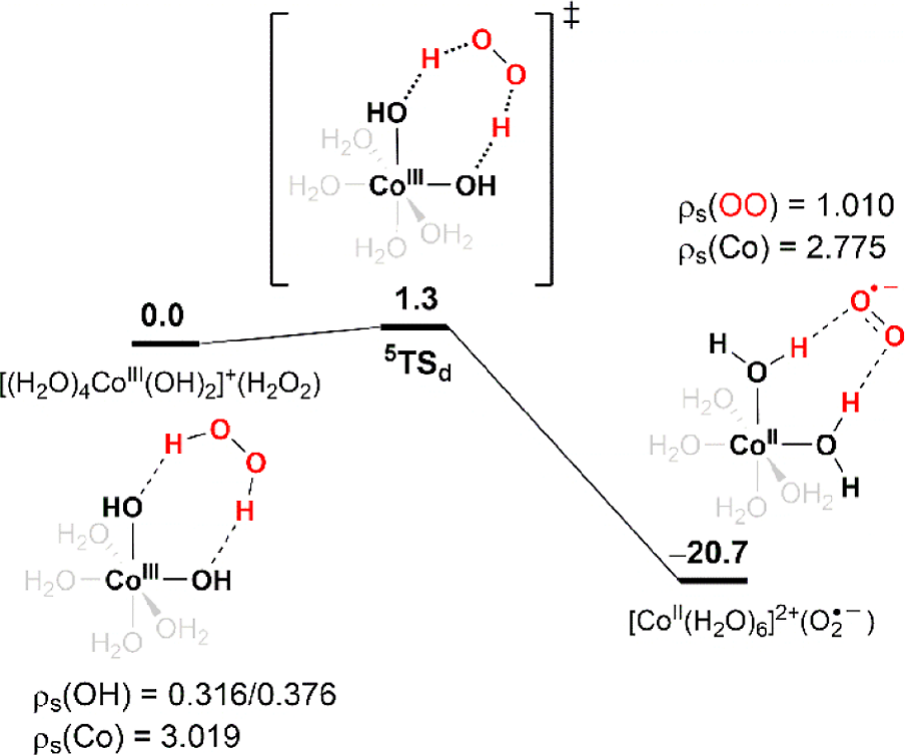
Free-energy profile for the reaction between [(H_2_O)_4_Co^III^(OH)_2_]^+^ and H_2_O_2_. The energy unit is kcal/mol.

Several studies have claimed that no Co(III) formation was observed during Co(II)-mediated H_2_O_2_ activation.^31,41,48^ According to our DFT calculations, because Co(III) species possess from moderate to strong oxidizing abilities and H_2_O_2_ is usually used in excess in experiments, a possible explanation for the previous observations is that Co(III) species do form but readily oxidize H_2_O_2_, regenerating Co(II), and, therefore, do not accumulate. To test this hypothesis, the reaction between [(H_2_O)_4_Co^III^(OH)_2_]^+^ and H_2_O_2_ was calculated, revealing that, indeed, [(H_2_O)_4_Co^III^(OH)_2_]^+^ can efficiently return to [Co^II^(H_2_O)_2_]^2+^ by oxidizing H_2_O_2_ through a concerted proton-coupled hydrogen transfer mechanism, accompanied by superoxide radical formation (Figure 6).

## 5 Conclusions

The present DFT calculations suggest three reaction pathways for [Co^II^(H_2_O)_6_]^2+^-mediated H_2_O_2_ activation involving two H_2_O_2_ molecules. The proposed pathways account for the experimentally observed formation of reactive intermediates ^•^OOH/O_2_^•–^, Co(III) species (crypto-^•^OH), and ^1^O_2_. The finding that Co(III) species manifest from moderate to strong oxidizing capabilities and the confirmation of the existence of Co(III)–oxo/oxyl species in aqueous solutions are valuable for understanding and interpreting the experimental results of Co(II)-mediated AOPs. Another important finding is the possible roles of transient species HOOOH and Co(II)–OOOH in ^1^O_2_ generation during Co(II)-mediated H_2_O_2_ activation. To the best of our knowledge, the formation of these transient species has never been previously proposed in Fenton-like chemistry and awaits experimental validation. Because hydrogen trioxide exhibits considerably longer lifetimes in organic solvents than in water (with half-lives *t*_1/2_ of approximately minutes and milliseconds, respectively), we suggest to conduct [Co^II^(H_2_O)_6_]^2+^/H_2_O_2_ reaction in organic solvents to facilitate its detection. Because the proposed reaction pathway does not involve changes in the Co(II) oxidation state, it may also apply to other redox-inert metal ion systems and provides a practical strategy for selectively generating ^1^O_2_.

## Supporting Information Available

Linear regression of p*K*_a_; results of *trans*-[(H_2_O)_4_Co^II^(OOH)(H_2_O_2_)]^+^; comparison of high-spin and low-spin results; optimized coordinates (PDF)
DFT-coordinates(XYZ)

## Supplementary Material

ic4c03966_si_001.pdf

ic4c03966_si_002.xyz
