# Supplementary material for: Cobalt(II) Aqua Complex-Mediated Hydrogen Peroxide Activation: Possible Roles of HOOOH and Co(II)–OOOH Intermediates in Singlet Oxygen Generation
Source: Inorg Chem. 2024 Dec 25;64(1):554–62. doi: 10.1021/acs.inorgchem.4c03966 (PMC11734112; doi:10.1021/acs.inorgchem.4c03966)
Supplement: Supplementary file 1 — ic4c03966_si_001.pdf [file ic4c03966_si_001.pdf]

Supporting Information

Cobalt(II) Aqua Complex–Mediated Hydrogen Peroxide Activation: Possible Roles of HOOOH and Co(II)–OOOH Intermediates in Singlet Oxygen Generation

Hsing-Yin Chen* and Yu-Fen Lin

Department of Medicinal and Applied Chemistry, Kaohsiung Medical University, Kaohsiung 80708, Taiwan

E-mail: hychen@kmu.edu.tw

Table of Contents

- Page S2−S3: Linear regression between experimental pK_a and theoretical deprotonation energy (Figure S1, Table S1 and S2).

- Page S4: Free energy profile of decomposition of *trans*-$[(H_2O)_4Co^{II}(OOH)(H_2O_2)]^+$ (Figure S2).

- Page S5−S6: Comparison of high spin and low spin results (Figure S3−S5).

- Page S7−S17: Optimized *xyz* coordinates.

Linear regression of pK_a and ΔG_{-H} = G(conjugate base) – G(conjugate acid)

Table S1. SMD/TPSSh/Def2-TZVP calculated ΔG_{-H}[a] (kcal/mol) and experimental pK_a for aqueous transition metal ions.

Complex	ΔG_{-H}	Expt. pK_a
$[Fe^{III}(H_2O)_6]^{3+}$	265.8	2.2[a,b]
$[Cr^{III}(H_2O)_6]^{3+}$	270.3	3.7[a]
$[Sc^{III}(H_2O)_6]^{3+}$	274.1	4.3[a,b]
$[Cu^{II}(H_2O)_4]^{2+}$	280.5	7.5[a]
$[Fe^{II}(H_2O)_6]^{2+}$	285.8	9.4[a]
$[Co^{II}(H_2O)_6]^{2+}$	287.8	9.7[a,b]
$[Mn^{II}(H_2O)_6]^{2+}$	288.7	10.6[a,b]

[a]*J. Chem. Educ.* **1996**, *73*, 516. [b]*The Hydrolysis of Cations*. John Wiley & Sons, New York, **1976**.

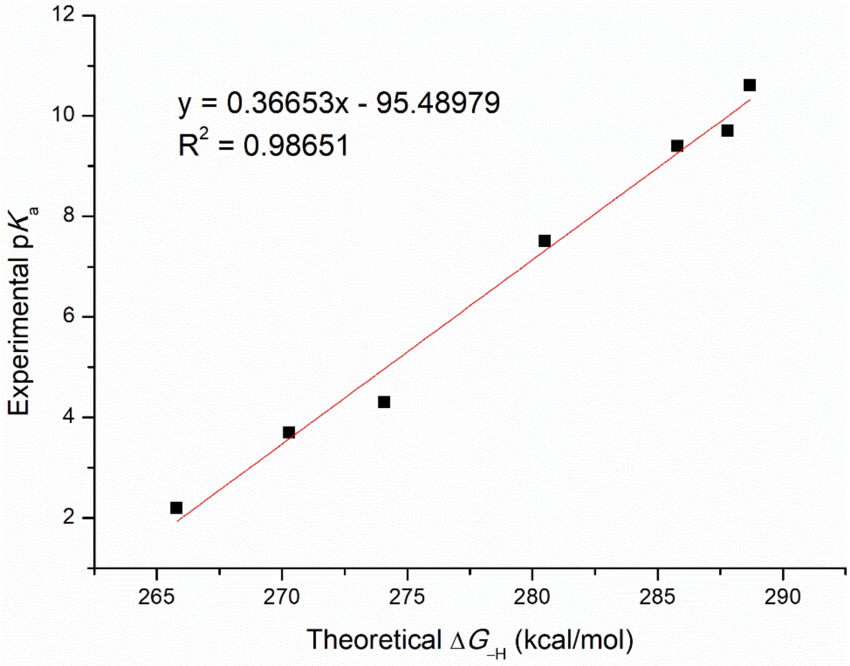

Figure S1. Plot of linear regression between experimental pK_a and theoretical ΔG_{-H}.

Table S2. Theoretical pK_a's estimated by using the linear equation pK_a = 0.36653ΔG_{-H} − 95.48979.

Complex	ΔG_{-H}	pK_a (theor.)	pK_a (expt.)
$[Ti(H_2O)_6]^{3+}$	264.5	1.4	1.3[a,b]
$[V(H_2O)_6]^{3+}$	266.4	2.2	2.3[a]
$[Mn(H_2O)_6]^{3+}$	258.3	−0.8	−0.6[a], 0.08[c]
$[(\mathbf{H}_2O)_5Fe^{III}(OH)]^{2+}$	275.2	5.4	5.3[d], 5.67[b]
$[Ni(H_2O)_6]^{2+}$	288.4	10.2	9.9[a,b]
$[V(O)(H_2O)_5]^{2+}$	274.8	5.2	5.3–6.0[a,e–h]

[a]*J. Chem. Educ.* **1996**, *73*, 516. [b]*The Hydrolysis of Cations*. John Wiley & Sons, New York, **1976**. [c]*Inorg. Chem.* **2006**, *45*, 10758. [d]*Atmos. Environ. Part A. Gen. Top.* **1990**, *24*, 79. [e]*J. Am. Chem. Soc.* **1953**, *75*, 6059. [f]*In Comprehensive Coordination Chemistry*; Wilkinson, G., Gillard, R. D., McCleverty, J. A., Eds.; Pergamon Press: New York, 1987; Vol. 3. [g]*J. Chem. Soc., Dalton Trans.* **1973**, 1156. [h]*Acta Chem. Scand.* **1955**, *9*, 1177.

Decomposition pathway of *trans*-[$(H_2O)_4Co^{II}(OOH)(H_2O_2]^+$

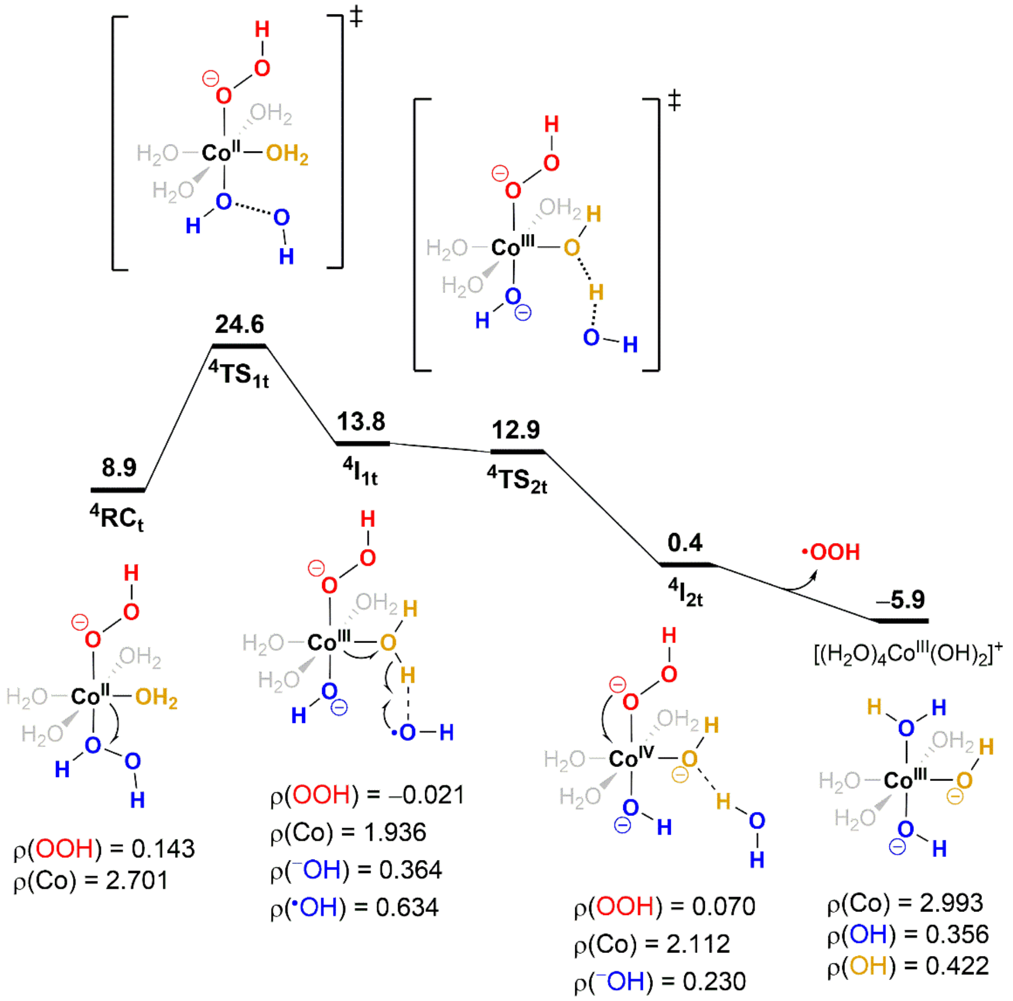

Figure S2. Free energy profile of decomposition of *trans*-[$(H_2O)_4Co^{II}(OOH)(H_2O_2)]^+$ (**RC$_t$**). Population analysis of spin density indicates that the HO–OH bond cleavage is triggered by an electron transfer from Co(II) to H_2O_2, leading to the formation of [$(H_2O)_4Co^{III}(OOH)(OH)]^+/^{\bullet}OH$ (**I$_{1t}$**). The leaving $^{\bullet}$OH immediately abstracts a hydrogen atom from water ligand to form Co(IV) species [$(H_2O)_3Co^{IV}(OOH)(OH)_2]^+/H_2O$ (**I$_{2t}$**). This Co(IV) species is not stable and will be immediately reduced to Co(III) species by $^-$OOH ligand. The activation energy of this pathway is 24.6 kcal/mol, higher than those of decomposition pathways of *cis*-[$(H_2O)_4Co^{II}(OOH)(H_2O_2)]^+$.

Comparison of high spin and low spin results

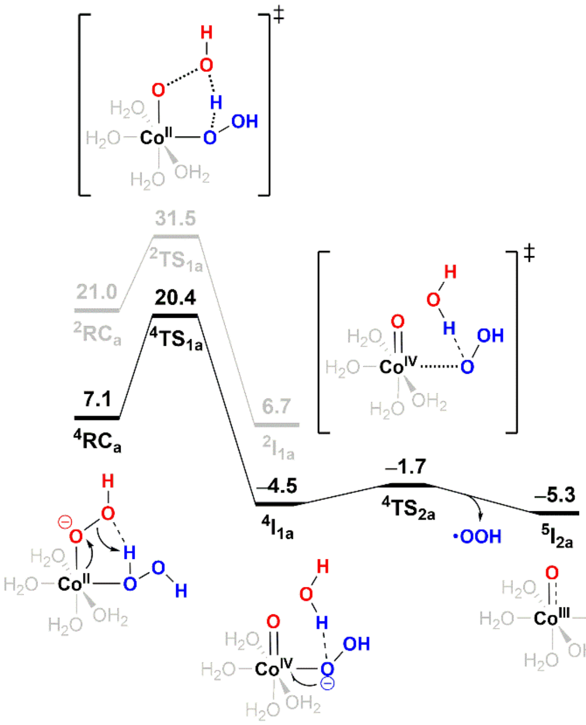

Figure S3. Free energy profile of decomposition of $[(H_2O)_4Co^{II}(OOH)(H_2O_2)]^+$ assisted by proton transfer from H_2O_2 to ^-OOH. The low spin doublet states are given in gray color.

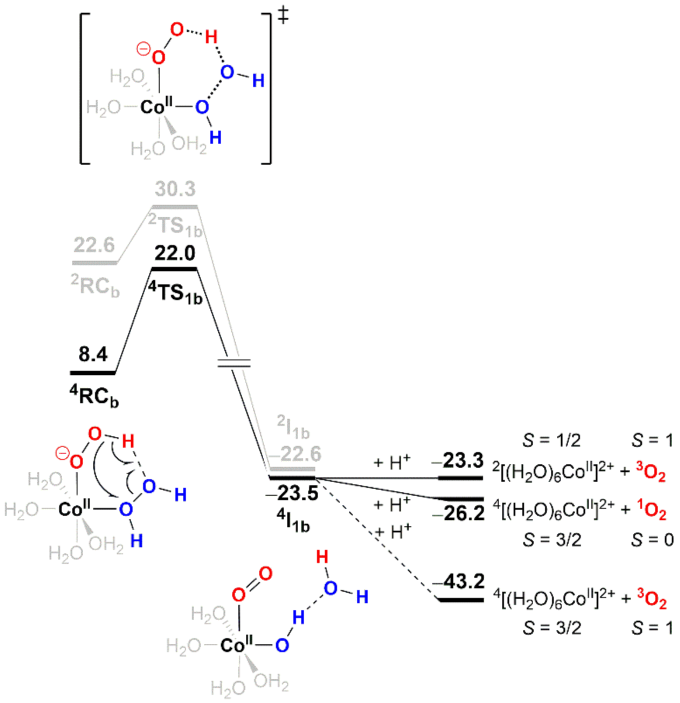

Figure S4. Free energy profile of decomposition of $[(H_2O)_4Co^{II}(OOH)(H_2O_2)]^+$ via hydrogen atom coupled electron transfer from ^-OOH to H_2O_2. The low spin doublet states are given in gray color.

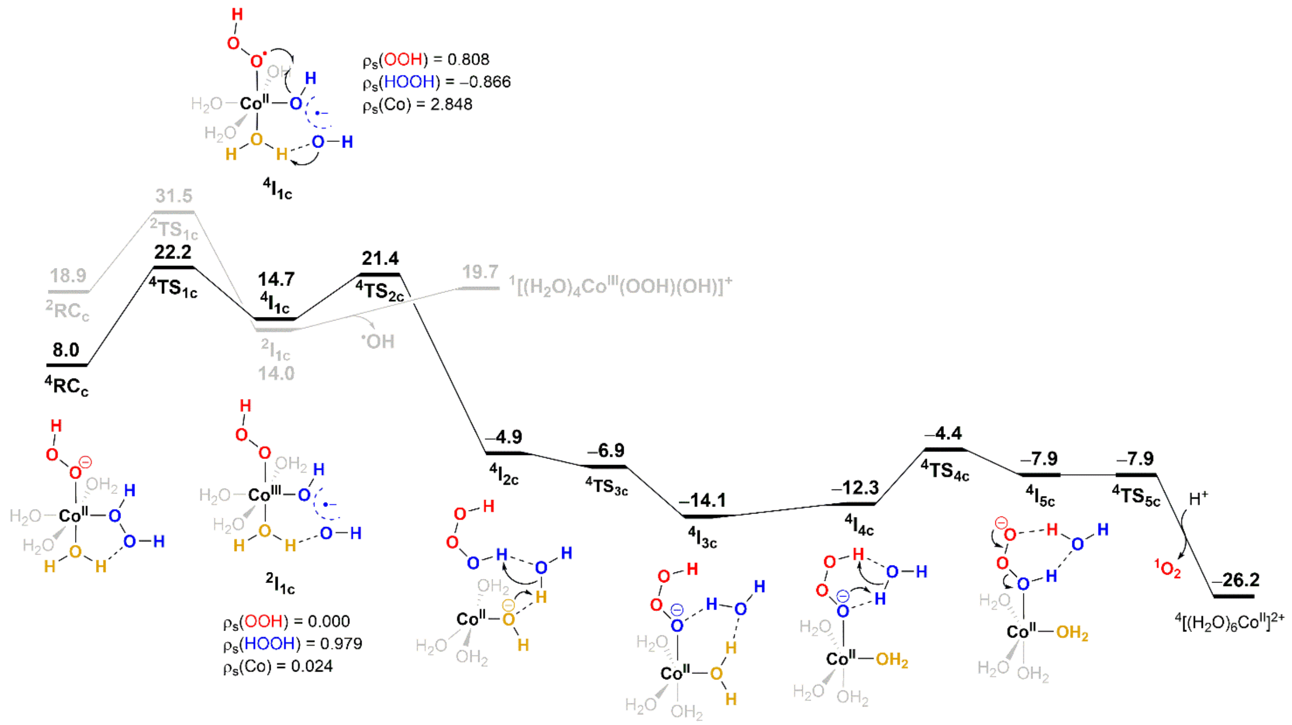

Figure S5. Free energy profile of decomposition of $[(H_2O)_4Co^{II}(OOH)(H_2O_2)]^+$ via electron transfer from ^-OOH to H_2O_2. The low spin doublet states are given in gray color. The calculations indicate that the mechanisms of high spin (quartet) pathway and low spin (doublet) pathway are different. While the high spin pathway involves the electron transfer from ^-OOH to H_2O_2, for the low spin pathway the decomposition of H_2O_2 is triggered by an electron transfer from Co(II) to H_2O_2. In addition, the reactant complex of the doublet state $^2\mathbf{RC_c}$ does not maintain octahedral six-coordinate structure; in fact, the H_2O_2 does not coordinate to Co(II) but locates at the second coordination sphere.

Optimized xyz coordinates

^{4}RC$_a$

1 4

O 0.62830400 0.26778300 2.09117600

H 0.77634700 -0.69819600 2.06904000

O 2.26098000 0.37483100 -0.38010700

H 2.60019100 -0.52856100 -0.29375500

O -0.08449300 2.45750900 -0.02855100

H -0.25927100 2.77378600 -0.92776400

O -1.75220200 -0.10734900 0.34943600

O 0.33830100 -2.02071900 -0.20169600

H -0.66573000 -2.06339700 -0.21943900

O -0.08808600 0.21406600 -2.31683500

H 0.38153100 -0.57260100 -2.63297300

H -0.23052000 0.36829600 2.52784900

H -0.89194800 2.66582000 0.46535700

H 2.63014700 0.85295500 0.37729300

H -1.01674600 0.05095300 -2.54093200

O -2.19701700 -1.41189300 -0.19844800

H -2.45238700 -1.17875800 -1.10622900

O 0.62539100 -2.44000400 1.16385800

H 1.29459900 -3.12764300 1.00597300

Co 0.08990900 0.28871800 -0.13528500

^{4}TS$_{1a}$

1 4

O 0.49807500 0.21018300 1.98772900

H 0.62659500 -0.76617900 1.99299700

O 2.15971800 0.10203600 -0.47028300

H 2.44882100 -0.61162500 0.11875800

O 0.16600100 2.40185300 -0.09275300

H -0.39820100 2.77271200 -0.78893200

O -1.74582200 0.05818100 0.03545400

O 0.11240300 -1.86262800 -0.20082700

H -1.07797400 -1.99298700 -0.08993600

O -0.10717700 0.44322600 -2.31201900

H 0.80904600 0.28838700 -2.59108900

H -0.32415800 0.36071500 2.47820400

H -0.21010800 2.72543800 0.74045800

H 2.55887500 0.90523900 -0.10218000

H -0.59414600 -0.33842300 -2.61645100

O -2.29328000 -1.93165900 0.04768800

H -2.66040200 -1.82751500 -0.84515200

O 0.60248900 -2.37006200 1.07801400

H 1.48642700 -2.68541300 0.82516800

Co -0.06988100 0.28411600 -0.12688100

^{4}I$_{1a}$

1 4

O 0.83161900 0.46357600 2.26032600

H 0.17708600 -0.03643200 2.77187200

O 2.04151700 0.36332000 -0.47498800

H 2.37954400 -0.52710600 -0.28510400

O 0.30023400 2.42338800 0.09537700

H -0.11467700 2.72612700 -0.72909100

O -1.53924500 0.39365500 0.53685000

O 0.16947800 -1.52388400 0.04144700

H -1.18153100 -1.99194400 -1.11338800

O -0.42920600 0.44821700 -2.08131800

H 0.42299700 0.26914100 -2.50641600

H 0.71137300 1.38475800 2.53848000

H -0.23933800 2.80304000 0.80750100

H 2.55013300 0.96370000 0.09338000

H -0.95026200 -0.38885100 -2.18275800

O -1.73066100 -1.96426500 -1.92896500

H -2.62705800 -1.77780900 -1.61531500

O -0.01463000 -2.18747000 1.27236500

H 0.85358400 -2.09416000 1.70534200

Co 0.03754800 0.36619000 0.14866400

^{4}TS$_{2a}$

1 4

O 0.97368100 0.58652900 2.23877900

H 0.23391800 0.31801100 2.80432500

O 2.05795700 0.39322800 -0.49214600

H 2.17694200 -0.48203700 -0.89240600

O 0.20291000 2.69548600 0.10571700

H -0.37340600 3.03291100 -0.59747700

O -1.59529000 0.32898700 0.69301200

O 0.17979800 -1.97757800 0.20232900

H -1.09217800 -2.24040200 -1.24574100

O -0.49001600 0.50865600 -2.02425000
H 0.35166300 0.48025800 -2.50281200
H 1.08947000 1.53110400 2.42350600
H -0.18200000 3.03222300 0.92935100
H 2.58171900 0.36880700 0.32380800
H -0.88546300 -0.39689200 -2.13731700
O -1.50699300 -2.02187100 -2.10343800
H -2.45498100 -1.97394900 -1.91388000
O -0.10766100 -2.52215200 1.38015600
H 0.51306900 -2.10871100 2.01986500
Co -0.02463800 0.56058300 0.14287800

$^{5}I_{2a}$

1 5
O 0.39714600 0.58937700 2.02985500
H 0.58072500 -0.32555900 2.29287900
O 2.19662500 0.24299000 -0.36082800
H 2.51721300 0.06591700 0.53655000
O 0.35582800 2.65382100 -0.17814700
H -0.18112100 3.01774200 -0.89847200
O -1.78167600 0.53143600 -0.00406100
O 0.17753400 -1.71969600 -0.12216200
O 0.12706000 0.51583100 -2.32842500
H 0.34442400 -0.39486600 -2.58003300
H -0.45082800 0.79764500 2.45136200
H -0.03395800 3.02078800 0.62995600
H 2.49697000 1.14464100 -0.55113200
H -0.76796100 0.65834400 -2.67168900
Co -0.09140400 0.50259000 -0.12927200
H 0.88735300 -1.88339700 -0.76191200
H -0.62137000 -2.07306500 -0.54135500

$^{4}TS_{3a}$

1 4
O 1.14529600 0.50874800 2.34319800
H 0.70008600 -0.20251600 2.82994400
O 2.17334200 0.42547500 -0.46674200
H 2.76554000 0.15074600 0.25224800
O 0.06439000 2.28970500 0.25717600
H 0.20036800 2.60175800 -0.65304600
O -1.34218500 0.08549400 0.75776100
O 0.48932800 -1.54469300 0.33302300
H -2.01382000 -2.20425000 -1.23536100
O -0.23423800 0.22081300 -1.89923000
H 0.57605900 -0.09453500 -2.32700400
H 0.73742200 1.32395500 2.67366600
H -0.87103600 2.46782400 0.45031500
H 2.39197500 1.35568100 -0.63770700
H -0.88700800 -0.52446100 -2.00168200
O -1.99601900 -1.82829800 -2.12808800
H -2.88749000 -1.47336300 -2.25865800
O -0.31751100 -2.10828300 1.31890100
H -1.03021700 -1.37263200 1.36320000
Co 0.24389900 0.26275200 0.24427700

$^{4}I_{3a}$

1 4
O 1.05758200 0.55334800 2.24658200
H 0.91554500 -0.30455900 2.67579300
O 2.13533100 0.45611400 -0.59213900
H 2.76053300 0.20229100 0.10437100
O -0.04965400 2.40012600 0.05203000
H -0.46059000 2.54941600 -0.81460300
O -1.59072600 0.08001700 0.70168100
O 0.51952200 -1.64168900 0.25970400
H -2.74216700 -1.39817200 -1.47978900
O -0.31874100 0.39858500 -1.97557800
H 0.45473300 0.06046600 -2.44998000
H 0.43559200 1.15242700 2.68652500
H -0.72906100 2.64402500 0.69987800
H 2.31873700 1.39151800 -0.77149500
H -1.04118700 -0.26283200 -2.14548700
O -2.31714200 -1.40752000 -2.35054300
H -2.97868700 -1.02860600 -2.94751600
O 0.28650900 -2.26106600 1.32153400
H -1.65557300 -0.72091100 1.24334700
Co 0.16955600 0.29478100 0.16632100

$^{4}RC_{b}$

1 4
O 0.31898400 -0.65169700 2.08126700
H -0.62808800 -0.91907900 2.17951200

O 2.09455300 -0.22638400 -0.38975100
H 2.27717900 -0.32725300 -1.33623600
O -0.02465300 1.91357100 -0.13336400
H -0.50359800 2.21272100 0.65413400
O -1.96445200 -0.36804200 0.29679200
O 0.20453300 -2.59591400 -0.39119400
H 1.00673200 -2.89603800 0.07239400
O -0.27930300 -0.17396100 -2.30321200
H 0.27815000 0.55474900 -2.61461300
H 0.37702200 0.22662200 2.48458900
H 0.89766900 2.16242900 0.02945300
H 2.48864500 -1.00634700 0.02848200
H 0.13108200 -0.97349700 -2.66530000
O -2.21241200 -1.30665800 1.41999100
H -1.93201400 -2.15714300 1.01885500
O -0.81036800 -3.43386500 0.22160700
H -1.11650500 -3.94650300 -0.54744200
Co -0.08328700 -0.29955200 -0.11076100

^{4}TS$_{1b}$

1 4
O 0.37423700 -0.53834700 2.02618800
H -0.48407400 -0.94506700 2.27053300
O 2.19350500 0.01996400 -0.42692700
H 2.32625900 -0.19867200 -1.36154400
O 0.01645800 1.77147500 -0.07678900
H -0.40676200 2.06783700 0.74361700
O -1.94350000 -0.44248000 0.41259800
O 0.30056000 -2.32853100 -0.26754300
H 0.75401800 -2.64593000 0.53230200
O -0.29173600 -0.40712700 -2.22989600
H 0.34195600 0.21090300 -2.62513500
H 0.33659300 0.36755400 2.36928300
H 0.96503500 1.92480300 0.05904000
H 2.63244800 -0.69585000 0.05650200
H -0.06507100 -1.27659500 -2.59351200
O -2.16870800 -1.50281200 1.37697400
H -1.84290000 -2.30482900 0.87763300
O -1.04665800 -3.48048300 -0.05493400
H -1.35518100 -3.37206800 -0.97143000
Co -0.09061100 -0.41458600 -0.09475700

^{4}I$_{1b}$

1 4
O 0.78167700 0.41352000 2.09449700
H 0.00903300 -0.04613900 2.46038600
O 2.38816100 0.66648300 -0.58425700
H 2.59653900 0.06051000 -1.31289600
O 0.14855700 2.40782200 -0.10360100
H -0.33964600 2.69656600 0.68325400
O -1.55263400 0.19098000 0.15716800
O 0.58888900 -1.47824400 -0.25525900
H -0.16660600 -1.99780100 0.08476000
O 0.00301000 0.59291000 -2.40124400
H 0.36567600 1.47165800 -2.58903300
H 0.66472400 1.33701100 2.36543900
H 1.02215300 2.82360000 -0.03617700
H 2.90614700 0.33759900 0.16738600
H 0.64109900 -0.02246200 -2.79251700
O -1.99294200 -0.23415700 1.25841900
H -2.24378700 -2.74452600 0.78571200
O -1.53361300 -3.35099700 0.53084100
H -1.79278400 -3.66368000 -0.34757200
Co 0.35251800 0.33132100 -0.12112800

^{4}RC$_{c}$

1 4
O -0.00752200 0.36434500 2.03890500
H -0.90882700 -0.04531700 2.01467800
O 2.16353700 0.34111400 -0.44045400
H 2.35134200 -0.53425800 -0.03986200
O -0.01029800 2.53350500 -0.23602800
H 0.10729700 2.83030000 -1.15106000
O -1.92478300 0.01861700 -0.12909400
O 0.41125400 -1.99723100 0.12118100
H -0.12236300 -2.21820500 0.90589800
O -0.12592100 0.32630700 -2.40665600
H 0.77033200 0.16963300 -2.74029300
H -0.15494100 1.28576000 2.29712800
H -0.90599100 2.81626700 0.00257500
H 2.50538900 0.98876100 0.19358900

H -0.62621400 -0.46320900 -2.66251700
O -2.26336600 -0.73309600 1.10490400
H -3.07457600 -0.28129100 1.38817800
O 1.76069400 -2.18783500 0.62387100
H 2.04158200 -2.97136900 0.11867500
Co -0.01426000 0.33219700 -0.20507700

^{4}TS$_{1c}$

1 4
O 0.13791500 0.28842600 2.00060900
H -0.79097200 0.04421500 2.19830700
O 2.20197000 0.36635400 -0.28972200
H 2.33363500 -0.52211700 0.11896600
O -0.06331900 2.37498200 -0.31153300
H 0.60918000 2.61142300 -0.96869200
O -1.95993900 0.16202000 0.05546200
O 0.40197800 -1.65156400 -0.32347400
H -0.26647400 -2.21010600 0.11352500
O -0.25944100 0.40327500 -2.38063700
H 0.63695900 0.63562800 -2.66845800
H 0.21454000 1.22490700 2.23843800
H -0.90485900 2.65373900 -0.70372800
H 2.45996800 1.00001300 0.39558700
H -0.40836300 -0.48555500 -2.73785800
O -2.32786600 -0.60580100 1.22958600
H -3.20062600 -0.23125000 1.43803600
O 1.82481100 -2.22373600 0.65368600
H 2.14600400 -2.80225000 -0.06015500
Co -0.08263900 0.19581200 -0.17237300

^{4}I$_{1c}$

1 4
O 0.18892400 0.39741200 2.05468500
H -0.69069800 0.23715400 2.43137800
O 2.08867900 0.15209500 -0.31475600
H 2.17173200 -0.84327000 -0.10864600
O 0.23482100 2.44352400 -0.18686700
H 0.90582200 2.61585800 -0.86485400
O -2.00801100 0.44908600 0.02461500
O -0.03115300 -1.73473400 -0.11683100
H -0.27121000 -2.02550100 0.77627000
O -0.20512000 0.24715700 -2.30505900
H 0.68903300 0.38329300 -2.65399300
H 0.37746400 1.32836400 2.24919700
H -0.57910300 2.83938100 -0.53373500
H 2.47704400 0.61172300 0.44352500
H -0.44584600 -0.65231400 -2.57492600
O -2.57164900 -0.27174600 1.03203500
H -3.50996600 0.00567500 1.01891600
O 2.02410000 -2.38593100 0.22296200
H 1.91217000 -2.82856100 -0.63350200
Co -0.05457400 0.25974600 -0.13484200

^{4}TS$_{2c}$

1 4
O 0.29499300 0.27987600 2.08747100
H -0.58057400 -0.09601900 2.29684600
O 2.24178700 -0.74327400 0.05415400
H 1.94684700 -1.74527800 -0.03703600
O 0.44225100 2.20058300 -0.20296100
H 0.12958300 2.48294200 -1.07582600
O -1.92812700 -0.80896800 0.23340200
O -0.45179800 -1.90555200 -0.09741400
H -0.40278700 -2.24398000 0.81269700
O 0.10569000 -0.04076400 -2.24270500
H 0.84311900 0.44407700 -2.64307200
H 0.23165300 1.21631500 2.32727800
H -0.16804900 2.60960400 0.42895700
H 2.57005300 -0.63665500 0.95807000
H 0.23558700 -0.96247200 -2.51360200
O -2.24492600 -0.81107700 1.58238700
H -2.83777600 -1.57881700 1.69017900
O 1.29635800 -3.07479400 -0.16954100
H 1.16232200 -3.22338900 -1.11696300
Co 0.31937300 0.06872500 -0.09659800

^{4}I$_{2c}$

1 4
O -0.05483500 0.88867700 1.93080300
H -0.76771900 0.21620700 2.01028200
O 2.04279100 -0.43038600 -0.19109200
H 1.34625600 -1.83428400 -0.23878900

O 0.43084300 2.83596600 -0.40111500
H 0.29601000 3.04947500 -1.33682900
O -2.29936400 -1.41111400 0.45540700
O -1.24390000 -1.01376400 -0.41384000
H -0.57133100 -1.78626100 -0.37834800
O 0.48113300 0.54492100 -2.32158200
H 1.31180600 0.05919000 -2.44499700
H -0.47815700 1.74367500 2.10008000
H -0.33627400 3.20964400 0.05904400
H 2.51505900 -0.35347600 0.64901400
H -0.21226300 -0.08895100 -2.56019400
O -1.90823200 -1.18701700 1.82475600
H -1.43862200 -2.01396500 2.04567600
O 0.74200700 -2.66399700 -0.26796800
H 0.93252400 -3.10230000 -1.10889400
Co 0.45809700 0.73322300 -0.10966000

^{4}TS$_{3c}$

1 4
O -0.19190700 1.03809500 2.01997800
H -0.68807400 0.21307400 2.21724800
O 1.98183000 -0.29096200 -0.00736500
H 1.52832800 -1.45764400 -0.22898800
O 0.13997200 2.70844100 -0.62160500
H 0.00215600 2.75623600 -1.57970900
O -2.01893300 -1.46065500 0.58296900
O -1.04000300 -1.00481600 -0.35050600
H -0.21677400 -1.79708600 -0.40493600
O 0.43568200 0.45579400 -2.31484200
H 1.13530700 -0.20672800 -2.41707900
H -0.83340300 1.75762500 2.11985300
H -0.61344000 3.16959000 -0.22316600
H 2.33690400 -0.28160900 0.89189100
H -0.36775700 0.01144700 -2.62373900
O -1.49321400 -1.39994800 1.92616300
H -0.95624000 -2.21282900 1.98024000
O 0.91834200 -2.44113100 -0.41716600
H 1.09948800 -2.73303400 -1.32171100
Co 0.22596700 0.70027600 -0.01186900

^{4}I$_{3c}$

1 4
O -0.32093800 0.53683300 1.96469300
H -1.03174600 -0.14123800 2.07007400
O 2.03881800 -0.70775100 -0.36460400
H 1.87221200 -1.61620000 0.00397000
O 1.18614100 2.13538100 -0.02642700
H 1.29566100 2.44048600 -0.93965800
O -2.22622600 -1.47262900 0.25484800
O -0.91626700 -1.48603300 -0.27459200
H 0.22264100 -2.64593200 0.32270000
O -0.08541300 0.56867800 -2.31479400
H 0.80527100 0.47045400 -2.68367500
H -0.74849000 1.39636500 2.09425000
H 0.63156400 2.80732700 0.39769200
H 2.67121800 -0.29038500 0.23823200
H -0.57046600 -0.20730200 -2.63444900
O -2.17145500 -1.44150400 1.73090900
H -1.88687100 -2.35017000 1.94085600
O 1.08456900 -3.05799200 0.60046800
H 1.22668500 -3.79088700 -0.01449900
Co 0.11067300 0.22958100 -0.08627100

^{4}I$_{4c}$

1 4
O -0.37070500 0.56074100 1.71457600
H -1.14407200 -0.04958600 1.81295700
O 2.27896800 -0.70218500 -0.41979600
H 2.50041400 -1.39260000 0.22300400
O 1.50413400 1.95299700 -0.16641900
H 1.25136200 2.49708000 -0.92631900
O -2.03930500 -1.65409500 0.07375500
O -0.64356900 -1.86215700 -0.10848000
H -0.57286000 -3.19451000 1.08896200
O -0.49858300 0.70223100 -2.00225200
H 0.19323200 1.11595000 -2.54007100
H -0.73460500 1.45784600 1.68929700
H 1.18227500 2.44378300 0.60352600
H 2.80162300 0.07376600 -0.16237000
H -0.83216200 -0.03424400 -2.53732600
O -2.30168200 -1.35204100 1.49856400

H -2.01601000 -2.20826000 1.90838500
O -0.92116300 -3.72925400 1.84565800
H -0.27003400 -3.61012700 2.55109700
Co 0.26156000 -0.09535600 -0.19678300

$^{4}TS_{4c}$

1 4
O -0.52252000 0.54363900 1.65641100
H -1.27548900 -0.12306200 1.74791300
O 2.21326400 -0.85214000 -0.40124600
H 2.41609900 -1.56890700 0.21886900
O 1.52104600 1.85428100 -0.06835300
H 1.22512900 2.44547000 -0.77577300
O -2.17903600 -1.65466400 0.18063900
O -0.77568900 -1.94558100 -0.16236500
H -0.58058300 -2.69623300 0.70984300
O -0.47260900 0.66217000 -1.94552800
H 0.23315900 1.07822300 -2.46314600
H -0.92966700 1.41428700 1.54088600
H 1.23750500 2.28951600 0.74891100
H 2.79703300 -0.11787000 -0.15386000
H -0.81326600 -0.05317800 -2.50428300
O -2.25945000 -1.41613900 1.60237900
H -1.61034700 -2.38228900 1.93963400
O -0.79477700 -3.30043700 1.82048800
H -0.05542100 -3.09197000 2.40891800
Co 0.25443600 -0.16513800 -0.15036900

$^{4}I_{5c}$

1 4
O -0.55410500 0.57753600 1.62077300
H -1.34309300 -0.09137000 1.67668200
O 2.21844700 -0.83219100 -0.39297600
H 2.41477600 -1.55160000 0.22632000
O 1.53566300 1.87761500 -0.07293600
H 1.25027600 2.45421000 -0.79668800
O -2.27544600 -1.58888600 0.22398900
O -0.83188400 -1.90155000 -0.25270900
H -0.58794900 -2.58668800 0.42868700
O -0.45216800 0.67525300 -1.95382700
H 0.25366500 1.08238900 -2.47842100
H -0.92872000 1.46072300 1.49368500
H 1.23793500 2.32807000 0.73100700
H 2.81409400 -0.10612400 -0.14979700
H -0.79370400 -0.04896200 -2.50055700
O -2.27305200 -1.28953300 1.57742300
H -1.42646300 -2.70087500 2.10398100
O -0.79947100 -3.46737700 1.98556500
H -0.05274300 -3.26841800 2.56751900
Co 0.27196100 -0.12504300 -0.14295500

$^{4}TS_{5c}$

1 4
O -0.51914500 0.54472400 1.63197600
H -1.34249600 -0.03403800 1.70842600
O 2.25809100 -0.78858000 -0.39282800
H 2.47910200 -1.49250300 0.23554700
O 1.51919300 1.86177900 -0.10429300
H 1.18890100 2.45847400 -0.79156500
O -2.36256200 -1.51159600 0.24974700
O -0.75126800 -1.91124400 -0.32773100
H -0.55261100 -2.55333400 0.39404500
O -0.46545700 0.65090400 -1.97326300
H 0.23793300 1.06914500 -2.49220400
H -0.82346700 1.45883600 1.53773000
H 1.29870700 2.30165900 0.72967500
H 2.80345100 -0.02681300 -0.14024500
H -0.79696600 -0.07265400 -2.52690400
O -2.36388000 -1.29200200 1.54865500
H -1.48035300 -2.80090400 2.13228900
O -0.80867200 -3.51064700 2.01153800
H -0.10217300 -3.28505600 2.63274300
Co 0.26168800 -0.16896800 -0.16857500

$^{2}RC_{a}$

1 2
O 0.41299900 0.31914000 1.78460500
H 0.36511500 -0.65780200 1.94325100
O 2.15396100 0.67010000 -0.39098000
H 2.45698800 1.43545600 0.12185800
O -0.13089600 2.68430200 -0.14566600
H -1.07376500 2.84228800 -0.30259900

O -1.68845800 0.17262400 -0.02960600
O 0.24168800 -2.64900000 0.38572800
H -0.56339800 -2.11134200 0.13603900
O 0.03145100 0.42262600 -2.25728100
H -0.08333000 -0.49548900 -2.54883300
H -0.38134800 0.68064200 2.20690100
H 0.00543000 2.93921500 0.77907300
H 2.38371800 0.87490500 -1.31062600
H -0.78715300 0.87139000 -2.52192400
O -1.95143400 -1.24450900 -0.35969300
H -1.98120200 -1.22672100 -1.33145800
O 0.27123100 -2.42389800 1.83186600
H 1.18072800 -2.70666800 2.02762700
Co 0.13706700 0.46605600 -0.22335200

$^{2}TS_{1a}$

1 2
O 0.59437200 0.12878800 2.02309300
H 0.61272900 -0.84684400 2.11471200
O 2.20117500 0.23763600 -0.32079600
H 2.59728700 0.34794900 0.55824300
O 0.04798400 2.24666800 -0.20296100
H -0.87778300 2.45945900 -0.39956900
O -1.52061400 0.05067300 0.14425900
O 0.22637700 -1.95346500 -0.13841900
H -0.82798000 -1.98796200 -0.23301600
O -0.01507500 0.17759000 -2.27250000
H 0.15895800 -0.72078000 -2.59344800
H -0.23065800 0.40467200 2.45045400
H 0.18845000 2.56925300 0.70107800
H 2.44799200 1.03516200 -0.81539200
H -0.96389100 0.31163600 -2.42353300
O -2.21210500 -1.58420400 -0.35219200
H -2.32850000 -1.31575900 -1.27767100
O 0.44262900 -2.51885800 1.18737800
H 1.26934500 -3.00938700 1.03740800
Co 0.17660600 0.13336900 -0.11915700

$^{2}I_{1a}$

1 2
O 0.29703700 0.60325700 1.85621900
H 0.43612400 -0.35326700 2.07597200
O 2.18453700 0.34695400 -0.28594100
H 2.56968300 -0.14291300 0.45782600
O 0.44068100 2.46210600 -0.20736700
H 0.03653000 2.77011700 -1.03579400
O -1.55669100 0.61518100 0.05933500
O 0.11043100 -1.39255600 -0.01744400
H -1.53975300 -2.06503900 -0.28209900
O -0.00653900 0.37313900 -1.99172200
H 0.46623100 -0.40639600 -2.33104600
H -0.62085200 0.79902900 2.12394300
H -0.06003100 2.90274400 0.49905900
H 2.54183400 1.24680600 -0.20555300
H -0.95504800 0.19792700 -2.13347900
O -2.40637100 -2.48966100 -0.47350100
H -3.05695700 -1.81173100 -0.24701500
O 0.52599500 -1.91709300 1.25615900
H 1.47323200 -2.09554500 1.11458600
Co 0.16188700 0.47101000 -0.06113700

$^{2}RC_{b}$

1 2
O 0.30643600 0.15635000 1.93609200
H -0.66148700 -0.05326700 1.99774100
O 2.31050000 0.31925400 -0.17969600
H 2.59670300 0.19078300 -1.09733200
O 0.41625500 2.63094500 -0.03972000
H -0.41491500 2.90633800 0.37505900
O -1.61161900 0.35785800 -0.08273700
O 0.24273100 -2.10997100 -0.39580100
H 1.00915300 -2.47213600 0.08325800
O 0.19179300 0.46360900 -2.15623400
H -0.62303000 0.01359200 -2.42955100
H 0.41505200 1.03202500 2.33717900
H 1.09734500 2.84369500 0.61569100
H 2.62848900 -0.46333600 0.29646900
H 0.06174600 1.39040300 -2.41129500
O -2.10981800 -0.39888100 1.08756500
H -1.89639000 -1.31956600 0.81863800
O -0.85551600 -2.78858900 0.27206200

H -1.18151900 -3.36749700 -0.43930000
Co 0.26981000 0.37675700 -0.10134700

$^{2}TS_{1b}$

1 2
O 0.34975300 -0.57325900 1.86597300
H -0.60827300 -0.75749000 2.03230600
O 2.20580800 -0.39493300 -0.25510400
H 2.48464800 -0.55340700 -1.17095100
O 0.30479000 1.75623300 -0.13693900
H -0.52492500 2.04219300 0.27523900
O -1.66886700 -0.34529500 -0.02161200
O 0.10729300 -2.42122700 -0.33421700
H 0.90294800 -2.79864400 0.08303500
O 0.02719100 -0.32019500 -2.16197300
H -0.79065000 -0.77502100 -2.42072200
H 0.51689300 0.31470400 2.21955900
H 0.99437800 2.00081700 0.49852300
H 2.57796100 -1.13121100 0.25554600
H -0.12272000 0.61281800 -2.38579000
O -2.12595900 -1.10142500 1.14677200
H -1.91894100 -2.03139600 0.87604100
O -0.98322900 -3.49446100 0.32407200
H -1.36486200 -3.78428300 -0.52252400
Co 0.18263400 -0.43535800 -0.14503200

$^{2}I_{1b}$

1 2
O 0.21245500 -0.00079300 1.63322100
H -0.77362300 -0.02327200 1.75438200
O 2.27726500 0.32848200 -0.19683800
H 2.62830100 0.77402800 -0.98548200
O 0.17135100 2.31053900 -0.06289400
H -0.53059400 2.52518500 0.57328200
O -1.59047600 0.25308700 -0.36510600
O 0.45813100 -1.48120600 -0.63866200
H -0.11165800 -1.97852100 -0.01916200
O 0.34721000 0.57199400 -2.21045700
H 0.21363400 -0.33602400 -2.54050000
H 0.52731400 0.78512700 2.11113200
H 0.99169600 2.63153600 0.34611200
H 2.42593500 -0.62429800 -0.34722200
H -0.41866100 1.08401400 -2.52216200
O -2.21643800 0.06739300 0.75890800
H -1.76602900 -2.27539900 1.48458300
O -1.23036500 -3.03393600 1.20808300
H -1.81959300 -3.54085400 0.63153400
Co 0.27600900 0.32040400 -0.27623200

$^{2}RC_{c}$

1 2
O 1.00166900 -0.96977700 -2.05546800
H 1.94911200 -1.07736600 -1.78412600
O -1.41494500 0.34769100 -1.03989700
H -2.14360600 0.03729300 -0.45174200
O 1.17535800 1.87580600 -0.94033100
H 0.96042300 2.48941700 -0.22222600
O 1.95698200 -0.78024400 0.52636300
O -2.90132500 0.08089100 1.98451900
H -3.09019600 -0.65490500 2.59428800
O -0.18338300 0.48892400 1.54550800
H -1.13030400 0.28461200 1.72748900
H 0.99979000 -0.30307700 -2.75896300
H 2.13942100 1.78563900 -0.90167500
H -1.55546200 -0.09565800 -1.88956000
H 0.32222500 0.03596500 2.23659800
O 3.05257800 -1.04436500 -0.43126300
H 3.50458700 -0.18431500 -0.45487500
O -3.39457200 -0.44907500 0.72935700
H -4.21732600 0.05962300 0.61329100
Co 0.41073400 -0.13968600 -0.29784000

$^{2}TS_{1c}$

1 2
O 0.44521500 -1.10190000 -1.51371200
H 1.37046200 -1.30872300 -1.22494500
O -1.59237100 0.99028100 -0.91233700
H -2.31097200 0.38517400 -0.60869700
O 1.30527700 1.68451000 -0.89410000
H 1.84517200 2.03600000 -0.16966500
O 1.44171000 -0.57340300 1.00642800
O -1.30222800 -1.19274100 0.67408900

H -1.20411700 -1.98540300 0.11571300
O -0.33469200 1.32078400 1.61327700
H -1.29292100 1.42753600 1.72639900
H 0.52568200 -0.58626700 -2.33168400
H 1.93350400 1.20401300 -1.45472000
H -1.52355800 0.85372300 -1.86988600
H -0.02927200 0.88178800 2.42307500
O 2.47056600 -1.13949700 0.13418300
H 3.03497100 -0.36871900 -0.04807200
O -2.94940800 -1.02465300 0.41842900
H -3.14348900 -0.65023300 1.29525400
Co 0.01925700 0.13950000 0.01238600

$^{2}I_{1c}$

1 2
O 0.16689800 -1.14913300 -1.37337900
H 1.12230400 -1.40609000 -1.28408000
O -1.55663200 1.00083500 -0.70931900
H -2.25908100 0.27096700 -0.47086000
O 1.16651900 1.47549600 -1.01971800
H 1.77775000 1.91179700 -0.40288500
O 1.57667400 -0.56584600 0.83232800
O -1.01370900 -0.93027900 1.01912100
H -0.94768200 -1.81547800 0.62397600
O 0.04714800 1.50551600 1.43749200
H -0.80428100 1.97446500 1.39612500
H 0.06544900 -0.73656300 -2.24782400
H 1.73611800 1.00034400 -1.64702300
H -1.49664600 1.04460500 -1.67592700
H 0.05705800 1.06172300 2.30263800
O 2.42564300 -1.23281600 -0.13051300
H 3.02609000 -0.52275600 -0.41852300
O -3.01254200 -0.90278300 0.06728500
H -3.33677900 -0.62587300 0.94003900
Co 0.07022000 0.17965400 0.01629300

$^{4}RC_{t}$

1 4
O -0.16882100 -0.88061200 -1.89142200
H -0.39766700 -1.82163800 -1.84999000
H -0.95810200 -0.45037700 -2.25346400
O 1.24263600 -1.97918900 0.66443800
H 0.73766300 -2.74843500 0.36068900
H 1.28157300 -2.07610100 1.62774800
O 0.62252800 0.61195800 2.14064800
H 1.35850500 0.10268900 2.51104200
H -0.11630300 0.46179900 2.74945000
O -1.12940200 1.60633800 -0.38701200
H -0.97446400 1.78054300 -1.32684000
H -1.95778400 1.06631400 -0.35481800
O 2.00979300 0.83153100 -0.48545600
H 2.19982800 1.46903200 0.22578400
O -1.65525800 -0.93653900 0.87423600
O -2.80883100 -0.43449800 0.08865100
H -2.76189700 -0.98309500 -0.71185200
Co 0.01761700 -0.21066400 0.18733000
O 1.83679100 1.70591100 -1.63042400
H 2.69343900 1.59558100 -2.07890500

$^{4}TS_{1t}$

1 4
O -0.15847500 -0.54302700 -1.94699400
H -0.40454500 -1.47983200 -1.89047000
H -0.95915400 -0.09118500 -2.25676900
O 1.38078500 -1.66969500 0.48762100
H 1.15102200 -2.29319600 -0.21896000
H 0.97433000 -2.04230100 1.28602800
O 0.90639600 0.82760600 1.95547900
H 1.18193600 0.06394600 2.48662200
H 0.10914100 1.16146500 2.39579600
O -0.92278200 1.90214500 -0.39451000
H -0.73972500 2.14928800 -1.31376400
H -1.77147500 1.40800200 -0.41931300
O 1.85125800 0.90458500 -0.62504300
H 2.54051800 0.99124700 0.05384400
O -1.28756800 -0.62273100 0.81639800
O -2.50724400 -0.22970500 0.12976200
H -2.52259700 -0.83282600 -0.63378700
Co 0.24316000 0.12347100 0.04100400
O 2.59461700 2.04979200 -1.79922300
H 2.64130200 1.36816100 -2.49130700

$^4I_{1t}$

1 4

O -0.58084900 -0.91484300 -1.81313300

H -0.82161600 -1.84683900 -1.69488600

H -1.41118800 -0.46965400 -2.04214400

O 0.88655400 -2.00196300 0.64917200

H 1.63789700 -2.14774800 0.05105400

H 0.24047700 -2.69081600 0.42318600

O 0.91357600 0.45840000 2.08682100

H 0.72217200 -0.24762000 2.72335100

H 0.35938600 1.20269100 2.36876100

O -0.67521400 1.63290800 -0.23036000

H 0.05230100 1.98995100 -0.86760500

H -1.48853500 1.51533600 -0.74585000

O 1.67644800 0.34675200 -0.64987800

H 2.22313800 0.76552400 0.03446700

O -1.44172900 -0.72642000 1.01526100

O -2.64483400 -0.34604800 0.36964300

H -2.80309700 -1.08586000 -0.24519000

Co 0.05031000 -0.18867400 0.16348400

O 1.35299600 2.29144800 -1.61294300

H 1.31124300 1.77064900 -2.43235100

$^4TS_{2t}$

1 4

O -0.56575800 -0.91109800 -1.84719200

H -0.82164800 -1.83824300 -1.72580300

H -1.38923800 -0.45391500 -2.07635300

O 0.84341800 -1.99717500 0.63086500

H 1.59697300 -2.17888800 0.04549800

H 0.17240000 -2.66115100 0.40222300

O 0.94245900 0.45689400 2.14112400

H 0.74901800 -0.26622000 2.75738500

H 0.36620900 1.18290700 2.42583200

O -0.62306400 1.59451600 -0.20885700

H 0.22870900 1.97578400 -0.98213300

H -1.46026600 1.46425500 -0.68058600

O 1.73206800 0.37346400 -0.59446100

H 2.19671200 0.93069700 0.05323900

O -1.44262600 -0.71418800 1.01022700

O -2.64432000 -0.34704100 0.35649900

H -2.77721800 -1.07529800 -0.27767100

Co 0.06567600 -0.16631100 0.17519600

O 1.22571300 2.23233300 -1.62035600

H 1.16421500 1.70585300 -2.43381400

$^4I_{2t}$

1 4

O -0.00092500 -0.88067000 -1.69565300

H -0.89462700 -1.24270800 -1.80694400

H 0.00181300 -0.00396000 -2.18071000

O 0.85519500 -2.15438400 0.69704500

H 1.73595700 -2.25830000 0.29997400

H 0.28280100 -2.79201200 0.23935900

O 0.64644400 0.00814700 2.64353200

H -0.22821600 -0.16620100 3.02445400

H 0.74187100 0.97189200 2.68684600

O -0.37991800 1.41947500 0.16911500

H -0.07031500 1.89957700 -1.71798800

H -1.35409600 1.34737000 0.27631500

O 1.89819500 0.25687700 0.00936600

H 1.86570100 1.07392200 -0.51677800

O -1.44572700 -0.92266900 0.77245300

O -2.55283500 -0.12415000 0.39937300

H -2.69288400 -0.35611700 -0.53902500

Co 0.19866500 -0.26234900 0.32976000

O 0.02160100 1.62776900 -2.65258100

H 0.93073100 1.86566700 -2.88705200

$^5[(H_2O)_4Co^{III}(OH)_2]^+(H_2O_2)$

1 5

O 0.24944100 -0.48454300 1.63373500

H 1.11533500 -0.84411100 1.87875200

O 0.83958300 1.35985300 -0.62511100

O -1.87303100 0.34011300 0.01717700

H -1.69937200 1.18155600 0.50648300

O -0.69641000 -2.38939100 -0.34093900

H -1.15042700 -2.39911700 -1.19870200

O 2.03945000 -1.38627700 -0.55171400

H 2.15935100 -1.49460900 -1.50807400

O -0.35602300 -0.67643100 -2.46706300

H -0.83920500 0.11657500 -2.74654100
H -2.21580800 0.60910000 -0.84877600
H -1.40306900 -2.40566900 0.32301300
H 0.68384600 1.58394900 -1.55644100
H 2.73831500 -0.77074200 -0.28088100
H 0.47928000 -0.64220300 -2.95771900
Co 0.15026900 -0.33509700 -0.25376900
O -0.02336200 2.02818300 2.39876300
H 0.06985800 1.04689600 2.17956800
O -0.70448400 2.53193700 1.21254900
H -0.05183300 2.30209800 0.49060300

^5TS_d

1 5
O 0.43219100 -0.10738500 2.04409800
H 1.16274500 -0.69158900 2.29843600
O 0.93969300 1.82538200 -0.26549600
O -1.72523800 0.58353900 0.20219300
H -1.70973200 1.45691200 0.62481200
O -0.21243200 -2.11553500 -0.08069300
H -0.65928100 -2.15572700 -0.94077200
O 2.44601100 -0.60370800 -0.17400100
H 2.55971300 -1.01839000 -1.04330800
O 0.04405900 -0.32033800 -2.16127700
H -0.43355600 0.47394400 -2.44668300
H -2.04511200 0.74354500 -0.69908100
H -0.90536100 -2.27467000 0.57806400
H 0.37880900 2.18945200 -0.96629200
H 2.93034400 0.23502600 -0.22950900
H 0.92210500 -0.23300400 -2.56275200
Co 0.38098700 -0.02069300 0.08682300
O 1.02688600 2.15538500 2.78111600
H 0.81834700 1.12841700 2.46823800
O 0.28969300 2.91858500 1.86279300
H 0.53755700 2.47500200 0.91846900

$[(H_2O)_6Co^{II}]^{2+}(O_2^{\bullet-})$

1 5
O 0.10179300 -0.44686000 1.71067000
H 0.90804000 -0.91679500 1.96563100
O 0.51210000 1.60919400 -0.51773200
O -2.16398700 0.15290100 -0.34134600
H -2.20344700 1.11522200 -0.23424400
O -0.59666800 -2.57751100 -0.45560400
H -1.10272500 -2.70980700 -1.27133900
O 2.03435500 -1.09647400 -0.50196800
H 2.19665500 -1.52116200 -1.35779900
O -0.27468600 -0.54652800 -2.57375800
H -0.43981800 0.35663900 -2.88420100
H -2.55293500 -0.01751300 -1.21233700
H -1.23541500 -2.72647600 0.25804000
H -0.13168200 2.06075800 -1.08180000
H 2.57229300 -0.29068700 -0.51714100
H 0.59074800 -0.78109900 -2.94196400
Co -0.06560800 -0.44991500 -0.39047800
O 0.24551700 1.93511800 2.75368700
H 0.18037800 0.48573900 2.11400400
O 0.43021800 2.83294300 1.78490500
H 0.46892600 2.08594000 0.38170900
